# Predictors of Uptake and Timeliness of Newly Introduced Pneumococcal and Rotavirus Vaccines, and of Measles Vaccine in Rural Malawi: A Population Cohort Study

**DOI:** 10.1371/journal.pone.0154997

**Published:** 2016-05-06

**Authors:** Hazzie Mvula, Ellen Heinsbroek, Menard Chihana, Amelia C. Crampin, Storn Kabuluzi, Geoffrey Chirwa, Charles Mwansambo, Anthony Costello, Nigel A. Cunliffe, Robert S. Heyderman, Neil French, Naor Bar-Zeev, VacSurv Consortium

**Affiliations:** 1 Karonga Prevention Study, Chilumba, Malawi; 2 Institute of Infection & Global Health, University of Liverpool, Liverpool, United Kingdom; 3 London School of Hygiene & Tropical Medicine, London, United Kingdom; 4 Expanded Programme for Immunisation Office and Preventive Services Office, Ministry of Health, Lilongwe, Malawi; 5 Chief of Health Services, Ministry of Health, Lilongwe, Malawi; 6 Institute of Global Health, University College London, London, United Kingdom; 7 Liverpool School of Tropical Medicine, Liverpool, United Kingdom; 8 Division of Infection & Immunity, University College London, London, United Kingdom; 9 Malawi-Liverpool-Wellcome Trust Clinical Research Programme, College of Medicine, University of Malawi, Blantyre, Malawi; Instituto de Higiene e Medicina Tropical, PORTUGAL

## Abstract

**Background:**

Malawi introduced pneumococcal conjugate vaccine (PCV13) and monovalent rotavirus vaccine (RV1) in 2011 and 2012 respectively, and is planning the introduction of a second-dose measles vaccine (MV). We assessed predictors of availability, uptake and timeliness of these vaccines in a rural Malawian setting.

**Methods:**

Commencing on the first date of PCV13 eligibility we conducted a prospective population-based birth cohort study of 2,616 children under demographic surveillance in Karonga District, northern Malawi who were eligible for PCV13, or from the date of RV1 introduction both PCV13 and RV1. Potential predictors of vaccine uptake and timeliness for PCV13, RV1 and MV were analysed respectively using robust Poisson and Cox regression.

**Results:**

Vaccine coverage was high for all vaccines, ranging from 86.9% for RV1 dose 2 to 95.4% for PCV13 dose 1. Median time delay for PCV13 dose 1 was 17 days (IQR 7–36), 19 days (IQR 8–36) for RV1 dose 1 and 20 days (IQR 3–46) for MV. Infants born to lower educated or farming mothers and those living further away from the road or clinic were at greater risk of being not fully vaccinated and being vaccinated late. Delays in vaccination were also associated with non-facility birth. Vaccine stock-outs resulted in both a delay in vaccine timeliness and in a decrease in completion of schedule.

**Conclusion:**

Despite high vaccination coverage in this setting, delays in vaccination were common. We identified programmatic and socio-demographic risk factors for uptake and timeliness of vaccination. Understanding who remains most vulnerable to be unvaccinated allows for focussed delivery thereby increasing population coverage and maximising the equitable benefits of universal vaccination programmes.

## Introduction

Malawi has proactively pursued the fourth Millennium Development Goal of reducing child mortality which it is expected to meet [1]. The early adoption of thirteen-valent pneumococcal conjugate (PCV13) and monovalent rotavirus (RV1) vaccines has been part of this strategy, with introductions on 12^th^ November 2011 and 29^th^ October 2012 respectively. PCV13 and RV1 are given according to World Health Organisation (WHO) recommended schedule at 6 and 10 weeks for doses 1 and 2, and 14 weeks for third dose PCV13 along with Pentavalent vaccine (diphtheria, pertussis, tetanus, *Haemophilus influenzae* type B and hepatitis B) and oral polio. Initial catch-up vaccination for PCV13 was conducted at the time of introduction with infants <1 year of age at date of first dose to receive 3 doses at 1 month intervals, even if subsequent doses would be given in the second year of life. Currently no PCV booster is scheduled. Measles vaccine (MV) is currently given as a single dose at 9 months of age, but a number of African countries including Malawi are planning introduction of a second dose [2]. Nationwide and district level MV campaigns are conducted when necessary. In our study site in Karonga district there have been four MV campaigns among infants <1 year of age between 2011 and 2014; one in 2011 and three in 2014.

Vaccination remains the cornerstone of public health intervention to reduce childhood morbidity and mortality, but oftentimes there are select groups that achieve poorer coverage than the national average [3]. Furthermore, vaccine coverage estimates do not reflect timeliness of vaccination, which may frequently be delayed [4, 5]. Previous work in this setting prior to introduction of PCV1 and RV1 showed high uptake of vaccines but delays in schedule [6]. As part of a national evaluation of impact and effectiveness of new vaccines in Malawi [7], we analysed data from a population-based birth cohort study to investigate factors affecting vaccination coverage and timeliness in northern Malawi during the period of introduction of pneumococcal and rotavirus vaccines. We also took the opportunity to examine predictors of MV coverage and timeliness with a view to informing national considerations of introduction of a second dose.

## Materials and Methods

### Study population and design

**Fig 1:**
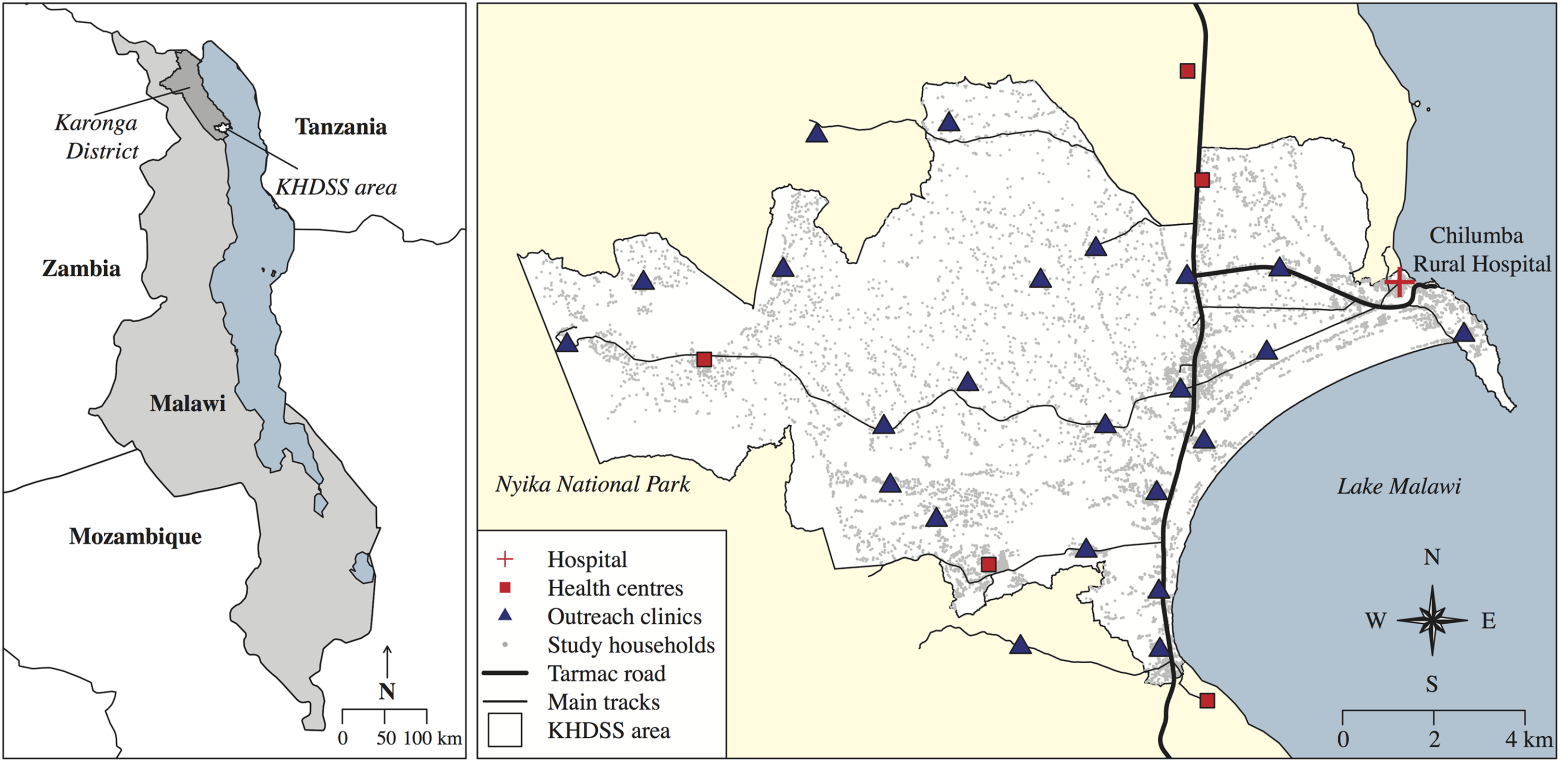
Map of the Karonga Health and Demographic Surveillance site (KHDSS), showing Chilumba Rural Hospital, 5 local health centres and 23 outreach clinic sites included in the study. Outline of Malawi and neighbouring countries downloaded from thematicmapping.org [10].

In this prospective population-based birth cohort study we followed up all children born in the Karonga Health and Demographic Surveillance Site (KHDSS) on or after: 11^th^ November 2010 (thus eligible for PCV13 catch-up), 30^th^ September 2011 (eligible for routine PCV13 schedule) and 17^th^ September 2012 (eligible for RV1). Established in 2002, the KHDSS situated in northern Malawi covers an area of 135km^2^ and has a population of 35,000 persons under continuous surveillance through which all births, deaths and migrations are recorded [8, 9]. The KHDSS area is predominantly rural, with agriculture, fishing and petty trading as main sources of income [8]. The surveillance area includes one rural hospital, five health centres and 23 outreach vaccination clinics (Fig 1). Data were collected during the KDHSS annual census at which all households are visited and individual and household socio-demographic data are collected by trained interviewers. Individual socio-demographic data including vaccine status for children <5 years were available until August 2014. Vaccine status and date of vaccination were transcribed from parent-held booklets (“health passports”) issued free by the government to all children at birth or first clinic visit. Absent vaccine documentation, parent/guardian reported vaccination status was recorded. Geographical Positioning System (GPS) coordinates were collected to calculate radial distance to nearest tarmac road or main track and to the nearest vaccination centre. We included in the analysis all children eligible for PCV13 or RV1 who were at least 1 year old at time of interview. We excluded: (1) children who died within the first year of life; (2) children who had migrated into the study area after 6 weeks of age from the PCV13/RV1 birth cohort or those migrating after start of the PCV13 catch-up campaign from the catch-up cohort; (3) any child whose date of birth or vaccination status could not be verified by written record. Children with documented evidence of vaccine receipt but lacking date were included in the coverage analysis, but excluded from timeliness analysis. Sensitivity analyses were performed to define risk factors associated with lack of written document and to repeat the main analyses including children without written documentation.

### Definitions

Individual uptake of vaccination was defined by the child’s written record in the health passport. Population vaccination coverage in the PCV13 and RV1 birth cohorts was calculated as the number of children receiving vaccination by one year of age divided by age-eligible population for each respective vaccine. For PCV13 catch-up campaign coverage we used the number of children vaccinated from among all catch-up eligible children in our study site at the time of data collection regardless of age. For measles vaccination we calculated vaccination coverage at one year of age, and vaccination coverage at any age. We were unable to distinguish measles vaccination as a result of mass campaign from routine doses given off schedule. Timeliness of vaccination for birth cohorts was calculated as number of days between the recommended vaccination age and the date vaccine was given, regardless of age. Children who never received the vaccine were right-truncated at one year of age. Delays in later doses subtracted any delays on account of prior doses (for example if dose 1 was given at 8 weeks (2 weeks delay) and dose 2 at 12 weeks, delay for dose 2 was considered 0, because it had been given correctly at 4 weeks after dose 1). Total delay was defined as the number of days between completing the schedule for a particular vaccine (third dose for PCV13, second dose for RV1, first dose for MV) and the recommended vaccination age. We presumed vaccine non-availability at local clinic, among the birth cohort only, when PCV13 or RV1 were administered later than the corresponding dose of Pentavalent vaccine.

### Statistical analysis

Potential individual level predictors of vaccine uptake and clinic level predictors of discordant delays (presumed non-availability) were separately examined using univariable and multivariable Poisson regression with robust standard errors [11, 12], and reported as risk ratios (RR) and adjusted RR (aRR) respectively (“risk” being probability of receiving a vaccine dose) [13]. We report timeliness as median days delay (and interquartile range [IQR]). Predictors of vaccine timeliness (total delay in vaccination) were examined using univariable and multivariable Cox regression, and reported as crude and adjusted hazard ratio (HR and aHR) respectively, where HR<1 implies delayed vaccination compared with baseline group (“hazard” being probability of receiving a vaccine dose at time *t*). Although our analysis examined numerous covariates, we did not perform correction for multiple comparisons in univariable analysis [14], but for multivariable models included initially only covariates achieving *P*-value <0.2 in univariable analysis. We retained in the final multivariable model those covariates achieving a likelihood ratio test *P*-value <0.05. Analysis was performed using Stata 12.1 (Statacorp, Texas) and R 3.0.1 (R Foundation for Statistical Computing, Vienna).

### Ethics

Informed written consent was obtained from all parents or legal guardians of participants. Ethical approval was obtained from the Malawi National Health Sciences Research Committee (#837) and the institutional review board of the London School of Hygiene and Tropical Medicine (#6047). The study was funded by the Wellcome Trust who had no input into data collection, analysis, interpretation or decision to submit for publication.

## Results

**Fig 2:**
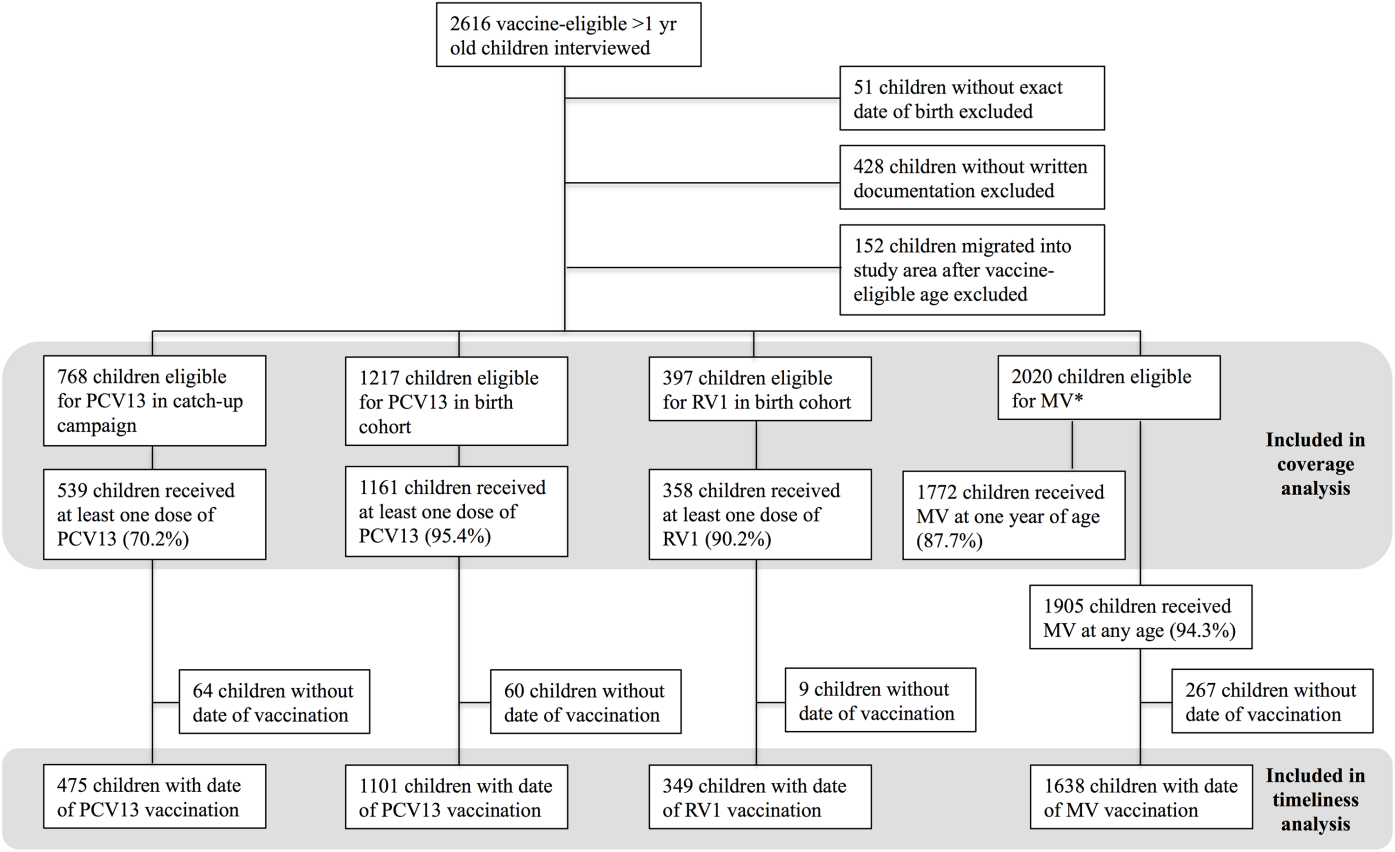
Flowchart of eligible children included in vaccination coverage and timeliness analyses. * There were 35 children for whom written documentation was available for MV but missing for PCV13/RV1.

We visited 2616 vaccine-eligible children at home who were >1 year at time of interview. Of these 51 had no documented birth date, 428 had no written documentation to confirm vaccination status and 152 migrated into the study area after vaccine-eligible age, leaving 1985 (75.8%) for analysis. Among these, 768 were eligible for PCV13 catch-up, 820 for PCV13 by routine schedule prior to RV1 introduction and 397 for both PCV13 and RV1 (Fig 2).

### Vaccination coverage

PCV13 coverage in the catch-up cohort for dose 1, 2 and 3 was 70.2% (539/768), 61.6% (470/763) and 49.8% (378/759), respectively. Among children birth-eligible for PCV13, coverage by one year of age for each of three doses was 95.4% (1161/1217), 94.4% (1149/1217) and 89.4% (1086/1215), respectively. Among those eligible for RV1, 90.2% (358/397) received one dose and 86.9% (344/396) received two doses. Coverage for MV by one year, regardless of routine dose or mass campaign, was 87.7% (1772/2020). Coverage for MV at any age was 94.3% (1905/2020).

### Vaccine ‘non-availability’

In the PCV13 birth cohort there were 276 of 1007 (27.4%) children who received the third dose of PCV13 later than the third dose of Pentavalent vaccine. Of 336 children eligible for RV1, 122 (36.3%) children received second dose RV1 later than the respective Pentavalent dose (S1 Table). Assumed “vaccine non-availability” for PCV13 decreased with time since national introduction, but vaccine non-availability for RV1 remained high and no clear association with time since introduction was observed. PCV13 non-availability was higher in the rainy than in the dry season (dose 1: aRR 1.63, 95% CI 1.12–2.37). Non-availability of vaccines was associated with further distance from the road for both PCV13 dose 1 (aRR1.57, 95%CI 1.07–2.30) and RV1 dose 1 (aRR 2.65, 95%CI 1.68–4.19). Children whose initial PCV13 doses were delayed compared with Pentavalent, were less likely to complete their PCV13 series (aRR 0.82, CI 0.76–0.89 where dose 1 delayed; aRR 0.86, CI 0.81–0.91 where dose 2 delayed) (S2 Table).

### Predictors of vaccination coverage by catch-up campaign

Among children in the PCV13 catch-up cohort, age at onset of campaign was strongly associated with uptake of third dose of PCV13 with 77.5% vaccinated among those <4 months old versus 9.7% amongst those 10–12 months old at time of campaign onset (aRR 0.13, CI 0.08–0.22). Children born to mothers aged ≥30 years were less likely to be vaccinated with three doses (aRR 0.77, CI 0.64–0.92). Vaccine coverage with at least one dose of PCV13 was higher amongst males (aRR 1.11, CI 1.02–1.20), but this effect was no longer observed for coverage with three doses (aRR 1.05, CI 0.93–1.18) (Table 1).

**Table 1:** Univariable and multivariable analysis of predictors of pneumococcal vaccine uptake in the catch-up campaign cohort.

Variable	N	Predictors of being vaccinated with one dose of PCV13			N	Predictors of being vaccinated with three doses of PCV13		
		Coverage (%)	RR (95% CI)	a RR^1^ (95% CI)		Coverage (%)	RR (95% CI)	a RR^2^ (95% CI)
Gender								
Female	372	65.9	1	1	367	47.7	1	1
Male	396	74.2	1.13 (1.03–1.24)	1.11 (1.02–1.20)	392	51.8	1.09 (0.94–1.25)	1.05 (0.93–1.18)
Child’s age at campaign onset								
<4 months	148	85.8	1	1	147	77.5	1	1
4–6 months	235	86.0	1.00 (0.92–1.09)	1.04 (0.57–1.88)	231	63.6	0.82 (0.72–0.94)	0.83 (0.73–0.95)
7–9 months	238	71.4	0.83 (0.75–0.92)	0.42 (0.24–0.72)	236	43.6	0.56 (0.48–0.67)	0.58 (0.49–0.68)
10–12 months	147	27.2	0.32 (0.24–0.42)	0.06 (0.03–0.11)	145	9.7	0.12 (0.08–0.21)	0.13 (0.08–0.22)
Mother’s age (yrs)								
<20	125	75.2	1	1	121	63.6	1	1
20–29	408	71.6	0.95 (0.85–1.07)	1.00 (0.90–1.11)	406	50.3	0.79 (0.67–0.93)	0.87 (0.75–1.00)
30–39	209	68.4	0.91 (0.79–1.04)	0.95 (0.84–1.07)	205	44.4	0.70 (0.57–0.86)	0.77 (0.64–0.92)
≥ 40	26	38.5	0.51 (0.31–0.84)	0.65 (0.41–1.03)	27	22.2	0.35 (0.17–0.72)	0.51 (0.28–0.93)
Mother’s education								
<5 years primary	63	65.1	1	1	63	47.6	1	1
> = 5 years primary	492	67.6	1.04 (0.85–1.26)	0.97 (0.83–1.13)	487	47.6	1.00 (0.75–1.32)	0.82 (0.65–1.02)
Secondary / tertiary	212	77.8	1.20 (0.98–1.45)	1.11 (0.95–1.30)	208	55.8	1.17 (0.88–1.56)	0.97 (0.76–1.23)
Mother’s marital status								
Married	682	70.1	1	1	674	49.4	1	1
Unmarried^3^	84	71.4	1.02 (0.88–1.18)	1.03 (0.91–1.16)	84	53.6	1.18 (0.75–1.86)	1.08 (0.90–1.30)
Mother mobile phone								
No	490	71.8	1	1	485	49.5	1	1
Yes	75	68.0	0.95 (0.80–1.12)	1.01 (0.87–1.17)	73	42.5	0.86 (0.65–1.14)	1.00 (0.76–1.30)
Mother’s occupation								
Farming	710	70.0	1	1	701	48.9	1	1
Other	45	75.6	1.08 (0.91–1.28)	1.14 (0.95–1.35)	45	50.1	0.98 (0.72–1.33)	1.13 (0.82–1.56)
Orphanhood								
Both parents alive	749	70.4	1	1	740	49.9	1	1
Father died	12	58.3	0.83 (0.51–1.34)	0.82 (0.55–1.23)	12	50.0	1.00 (0.57–1.77)	1.09 (0.68–1.74)
Place of birth								
Health centre	668	71.3	1	1	662	50.6	1	1
Home / TBA / other	70	65.7	0.92 (0.77–1.10)	0.91 (0.78–1.07)	68	47.1	0.93 (0.71–1.21)	0.94 (0.75–1.19)
Housing standard								
1 (lowest)	127	68.5	1	1	126	50.0	1	1
2	280	68.9	1.01 (0.87–1.16)	1.02 (0.91–1.15)	275	50.6	1.01 (0.82–1.25)	1.06 (0.87–1.27)
3	151	68.2	1.00 (0.85–1.17)	0.99 (0.87–1.14)	150	46.7	0.93 (0.73–1.19)	0.96 (0.77–1.20)
4 (highest)	124	72.6	1.06 (0.90–1.24)	1.07 (0.93–1.22)	124	50.0	1.00 (0.78–1.28)	1.00 (0.80–1.26)
Household size								
<4	148	74.3	1	1	145	57.2	1	1
4–6	396	69.7	0.94 (0.84–1.05)	1.00 (0.91–1.11)	393	50.1	0.88 (0.74–1.04)	1.05 (0.88–1.25)
≥ 7	224	68.3	0.92 (0.81–1.05)	0.96 (0.86–1.08)	221	44.3	0.77 (0.63–0.95)	0.95 (0.78–1.17)
Number of children <5 years in household								
1	329	70.8	1	1	324	52.5	1	1
2	394	69.5	0.98 (0.89–1.08)	0.96 (0.88–1.05)	391	47.3	0.90 (0.78–1.04)	0.95 (0.83–1.09)
≥ 3	45	71.1	1.00 (0.82–1.23)	0.96 (0.81–1.14)	44	49.8	1.00 (0.74–0.35)	0.99 (0.76–1.31)
Distance to road (km)								
<1	579	71.0	1	1	571	49.6	1	1
1–1.5	125	67.2	0.95 (0.83–1.08)	0.93 (0.84–1.04)	124	52.4	1.06 (0.88–1.28)	1.04 (0.89–1.21)
≥ 1.5	64	68.8	0.97 (0.81–1.15)	0.92 (0.77–1.10)	64	46.9	0.95 (0.72–1.24)	0.89 (0.68–1.16)
Distance to clinic (km)								
<1	514	72.0	1	1	509	50.9	1	1
1–1.49	176	67.1	0.93 (0.83–1.05)	0.93 (0.84–1.03)	174	50.6	0.99 (0.84–1.18)	0.98 (0.85–1.13)
≥ 1.5	78	65.4	0.92 (0.77–1.07)	0.94 (0.81–1.08)	76	40.8	0.80 (0.60–1.07)	0.85 (0.66–1.09)
Moved house								
No	742	69.7	1	1	734	49.5	1	1
Yes	26	84.6	1.21 (1.02–1.44)	1.14 (0.98–1.31)	25	60.0	1.21 (0.87–1.69)	1.10 (0.74–1.38)

^1^ Adjusted for child’s age at onset of catch-up vaccination campaign and sex

^2^ Adjusted for child’s age at onset of catch-up vaccination campaign and maternal age

^3^ Never married/divorced/widowed

### Predictors of vaccination coverage by routine schedule

Table 2 summarizes the predictors of low uptake for all studied vaccines. Time since national introduction was an important predictor of vaccination coverage in the PCV13 birth cohort, with higher coverage >9 months compared to 0–3 months since introduction (aRR 1.09, CI 1.05–1.13; aRR 1.16, CI 1.09–1.23 for first and third dose respectively) (S2 Table). Uptake was lower if vaccination was due in the rainy season than in the dry season (aRR 0.97, CI 0.95–1.00 for dose 1). Maternal farming was associated with a lower uptake of PCV13 (aRR 0.96, CI 0.93–0.98 for dose 1, aRR 0.93, CI 0.89–0.98 for dose 3). Children from larger households or those with more children were more likely to receive PCV13. Children were less likely to be fully vaccinated for the third dose of PCV13 if distance to the nearest clinic was ≥1.5km (aRR 0.89, CI 0.81–0.98).

**Table 2:** Summary of multivariate analyses on predictors of vaccine non-receipt.

Vaccine	Dose	Predictors of vaccine non-receipt^1^
**PCV13—catch-up cohort**	**1**	- older age at onset of catch-up campaign
		- being female
	**3**	- older age at onset of catch-up campaign
		- born to mother ≥30 years
**PCV13—birth cohort**	**1**	- vaccination due soon after introduction
		- maternal farming
		- household size <4 persons
		- vaccination due in the rainy season
	**3**	- receiving dose 1 later than pentavalent vaccine
		- receiving dose 2 later than pentavalent vaccine
		- vaccination due soon after introduction
		- distance to nearest clinic ≥1.5km
		- maternal farming
		- living with <3 other children <5 years
**RV1**	**1**	- distance to road ≥1.5km
		- born to married mother
	**2**	- distance to road ≥1.5km
		- living with >3 other children <5 years
		- born to mother <40 years
**MV**	**1**	- distance to nearest clinic ≥1.5km
		- maternal farming
		- low maternal education
		- living with more children <5 years

^1^Predictors listed in descending order by strength of association. Full details available in S2, S3 and S4 Tables

For RV1 uptake was higher if mother was not married (aRR 1.08, CI 1.01–1.15 for dose 1) or if maternal age was ≥40 years (aRR 1.10, CI 1.00–1.21 for dose 2), but lower if distance to the nearest road was ≥1.5km (aRR 0.80, CI 0.69–0.93 dose 1; aRR 0.75, CI 0.63–0.90 dose 2) (S3 Table). In contrast to the PCV13 birth cohort, children were less likely to be vaccinated with RV1 if they lived in a household with more than three children <5 years of age (aRR 0.77, CI 0-59-1.00 dose 2).

Infants were less likely to receive MV if there were other children in the household (aRR 0.94, CI 0.91–0.97), if distance to the nearest clinic was ≥1.5km (aRR 0.91, CI 0.85–0.98) or if their mothers were farmers (aRR 0.92, CI 0.89–0.96). Children were more likely to be vaccinated if the mother attended post-primary education (aRR 1.09, CI 1.00–1.18) (S4 Table).

### Timeliness of vaccination

**Fig 3:**
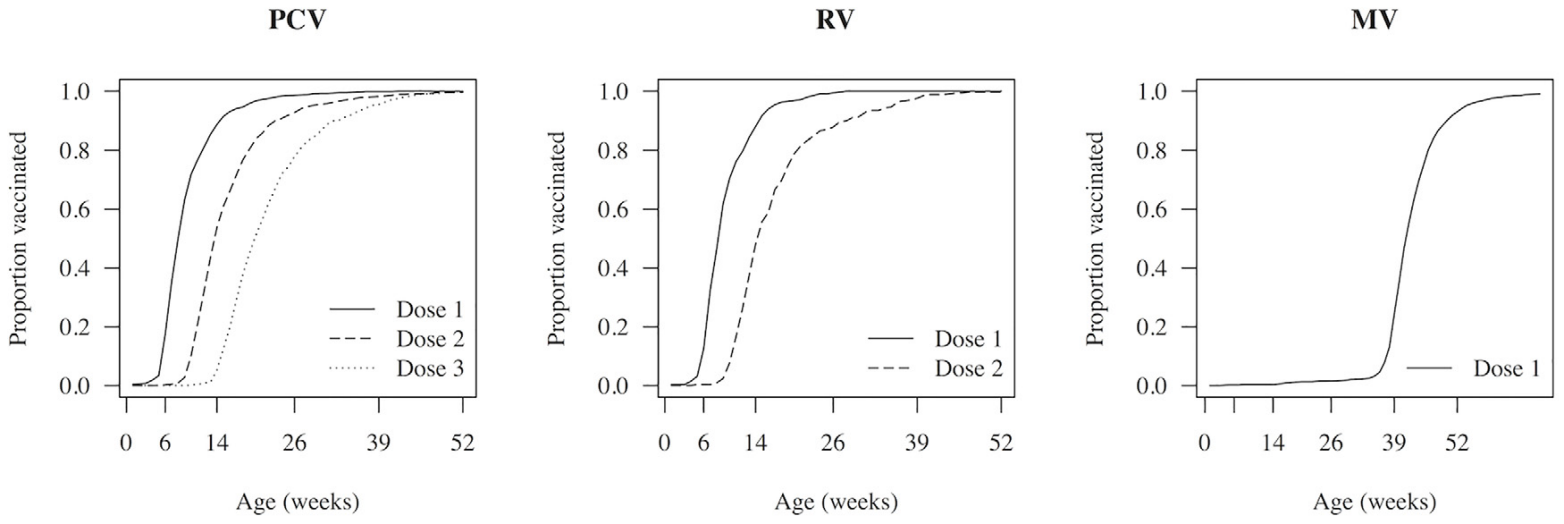
Timeliness of pneumococcal conjugate, rotavirus and measles vaccines, among vaccinated children in the Karonga Health and Demographic Surveillance Site.

Fig 3 shows the timeliness of vaccination in the eligible birth cohorts. Median time delay for each PCV13 dose was 17 days (IQR 7–36), 7 days (IQR 0–28) and 7 days (IQR 0–35) respectively. For RV1 doses, median delay was 19 days (IQR 8–36) and 7 days (IQR 0–35) respectively. MV median delay was 20 days (IQR 3–46). Table 3 summarizes the predictors for total delay in vaccination for all studied vaccines. Total delay in vaccination was associated with living further away from the road. Living with more under-five children was associated with delays for MV and RV1. Maternal farming and non-facility birth were associated with delays in PCV13 and MV. Lower maternal education was associated with delayed MV. PCV13 delay was associated with moving house in the vaccination period and vaccination due in the rainy season, but no such associations were observed for RV1 and MV (S5, S6 and S7 Tables).

**Table 3:** Summary of survival analyses on predictors of delay in vaccination.

Vaccine	Predictors of increased total delay in vaccination^1^
**PCV13**^2^	- moving house in the vaccination period
	- maternal farming
	- distance to road ≥1.5km
	- non-facility birth
	- vaccination due in the rainy season
**RV1**	- distance to road ≥1km
	- living with more children <5 years
**MV**	- maternal farming
	- low maternal education
	- non-facility birth
	- living with more children <5 years

^1^Predictors listed in descending order by strength of association. Full details available in S5, S6 and S7 Tables

^2^Delay calculated for PCV13 birth cohort only: not applicable to the PCV13 catch-up cohort

### Sensitivity analysis including children without written documentation

There were 428 children without written documentation of PCV13 and RV1 vaccine status. Verbal report of PCV13 vaccine status was available for 356 children, provided by the mother (44.9%), father (34.3%), a sibling (3.7%), other relative (16.9%) or a non-relative (0.3%). Coverage by verbal report amongst children without written documentation, excluded from the analyses reported above, was lower for the PCV13 catch-up cohort (45.8% (87/190) dose 1, 45.3% (86/190) dose 2, 45.3% (86/190) dose 3), PCV13 birth cohort (84.3% (140/166) dose 1, 84.3% (140/166) dose 2, 83.7% (139/166) dose 3) and RV1 (78.0% (39/50) dose 1, 76.0% (38/50) dose 2), but higher for coverage of MV (99.7% (361/362)). Moving house (aRR 0.89, CI 0.80–1.00) and being born to an unmarried mother (aRR 0.93, CI 0.87–1.00) were associated with lack of written documentation. Children born to farming mothers were found to be more likely to have written documentation available (aRR 1.17, CI 1.07–1.29). Including children without written documentation in the primary analyses did not change our main findings, although significance was lost for some risk factors, possibly as a result of misclassification of verbal report of vaccination status. No new risk factors for vaccine uptake were identified in analyses including only children without written documentation.

## Discussion

Malawi has been proactive in the trialling, introduction and post-roll out evaluation of vaccines, and as our data show, has achieved high coverage and good timeliness, even in our remote study setting. As with all universal programmes some gaps remain. Studies that have examined factors associated with vaccination coverage and timeliness of vaccines in sub-Saharan Africa, have found that lower vaccine uptake and untimely vaccination were associated with low socioeconomic status, low maternal education, non-facility birth, and increased distance to a health facility [4–6, 15–31] (S8 Table), consistent with the findings of this study. No studies were conducted in the context of newly introduced PCV13 and RV1. In this study located at a Demographic Surveillance Site, despite our communal context of homogenous cultural affiliation and of socioeconomic standing and remoteness, we found programmatic and socio-demographic characteristics that are associated with vaccination coverage and timeliness among *individual* infants in a rural region of Malawi. The recognition that there exist individual vulnerabilities even in an otherwise homogenous setting is important, and we return to this point in our recommendations.

We made the assumption that if Pentavalent vaccine was received on time but PCV13 was delayed then this was due to local non-availability of the latter. At the time of PCV13 introduction in late 2011 Malawi suffered major fuel shortages that impacted on distribution of newly introduced vaccines and many other societal functions. Although we are unable to verify this assumption, presumed non-availability reduced with time since national introduction and was associated with seasonality and distance from the road. More remote health centres generally serve a more scattered population residing away from dense centres, and often away from sealed roads. Such health centres are more difficult to access by the vaccine programme delivery mechanisms; we tried to capture this, albeit imperfectly, by including in distance to sealed road. Non-availability of early doses predicted not only total vaccination delay, but also non-completion of the vaccine course. Recognising the challenges in place at the time of PCV13 introduction, the Malawi National Immunisation Technical Advisory Group conducted several sessions to review lessons learnt from PCV13 in planning for RV1 introduction. These reviews included wide representation from ministerial, non-government and academic health planners with vaccine programme experience, epidemiologists, procurement, media, finance and transport specialists. Separately conducted coverage estimates from southern Malawi do suggest rapid attainment of RV1 population coverage [32], although in our more remote setting RV1 non-availability seemed to persist over time.

Despite the decision to provide catch-up vaccination to infants, the country was provided with doses adequate only for the birth cohort [33]. Coverage achieved among infants was moderate, but was low amongst older infants (9.7% for dose 3 amongst those 10–12 months at time of campaign onset). It is likely that available PCV13 doses were prioritised for younger infants, but alternative hypotheses for the lower uptake amongst older infants are that mothers were unaware of the programme for older infants, or that mothers were reluctant or could not afford to make an additional visit to the health centre, but accepted vaccination as part of the routine schedule for younger infants.

We identified several socio-demographic predictors of vaccine coverage and timeliness. Specific associations varied by vaccine and dose and should be interpreted with caution, particularly when results are counterintuitive, conflicting, of borderline significance or so small as to be not meaningful for example RV1 uptake being higher among unmarried mothers, or crowded households being associated with higher uptake of PCV13 coverage but lower uptake of RV1 and MV. Despite these caveats, we did find that infants living further away from the road or clinic were consistently more likely to be vaccinated late and less likely to complete vaccination as previously found in the same setting [6]. Both measures of distance capture slightly different challenges in reaching vaccine services. Distance from road is a measure of remoteness and in this setting a marker for socioeconomic status. Distance to clinic reflects accessibility more directly. Lower maternal education was consistently associated with lower vaccine uptake and more delays, although significance on multivariable analysis was only retained for MV. This finding provides further evidence for the importance of maternal education in remote rural settings, and of community sensitisation methods other than printed media. Infants born to farming mothers were more likely to be vaccinated late and less likely to complete vaccination for PCV13 and MV. It is plausible that farming mothers have fewer opportunities to bring their child to the clinic on time. With regards to the catch-up campaign for PCV13, greater maternal age was associated with lower uptake. For both RV1 and MV living in a household with multiple children <5 years was a risk factor for both lower uptake and delayed timeliness. Infants born outside of health facilities were vaccinated later for PCV13 and MV. For PCV13, vaccination due in the rainy season was found to be associated with lower uptake and more delays. In rural Malawi health facilities are spaced approximately every 10km to make access possible by foot. However, during the rains this is challenging, since torrents frequently wash away bridges and render footpaths impassable and farming communities are busy with planting and weeding. Noting the factors associated with MV coverage is important because if our coverage levels reflect those in the whole population <5 years herd protection against measles will not be achieved.

Vaccination timeliness is important for several reasons. Optimising vaccine schedules requires balancing benefits of delayed vaccination, such as prolonged immunity, against protection at an early age in the context of high force of infection [34–36]. Timeliness is also a marker of the functionality of the national vaccine delivery system so is of inherent interest to health service planners.

### Limitations

Our study has limitations. Excluding children if written documentation of dates was unavailable may lead to an overestimation of vaccine coverage for PCV13 and RV1 since reported coverage was lower among children without documentation. Coverage estimates for measles may have been underestimated if doses given during district-wide campaigns were unrecorded, consistent with higher coverage reported by parents than documented in health passport. A sensitivity analysis including children without written documentation did not alter the main findings of this study. Our data could not distinguish whether written documentation was missing because the health passport was truly lost, or because the mother, generally in charge of keeping the children’s health passports, was not available at time of the interview. The low proportion of mothers available at time of interview for children for whom written documentation was not available (44.9% vs. 84.3% for children with documentation) suggests that both scenarios occur. We included only children surviving to one year of age. We hypothesise that children dying in infancy are more likely to be unvaccinated, and this is currently being investigated [7]; results are anticipated in 2016. Our data come from an area under continuous demographic surveillance which may have an increased vaccine uptake. Although vaccine uptake may be higher in our Demographic Surveillance Site than in other areas, the risk factors we found for low uptake or poor timeliness of vaccination are likely to be relevant to other rural African settings not under continuous demographic surveillance. *A fortiori*, if in an area with relatively good service provision we identified children whose circumstances adversely affect vaccine coverage, then in other rural areas with similar socio-demographics but less functioning health systems, such children are very likely to be under-served. In the absence of reliable data on stock-outs, we made the assumption that delay in new vaccines when older vaccines were given on time was due to non-availability of new vaccines. The validity of this assumption cannot be confirmed. Our definition would miss non-availability of all vaccines, however in practice initial post-introduction delivery to clinics of PCV13 and RV1 occurred separately from routine vaccine delivery.

### Conclusion

Although vaccination coverage is moderately high in this rural population of northern Malawi, we found that infants born to lower educated mothers or farming mothers and those living further away from the road or clinic were at greater risk of being not fully vaccinated and being vaccinated late. We also found delays in vaccination to be associated with non-facility birth. Vaccine stock-outs which were more likely during the rains resulted in both a delay in vaccine timeliness and in fewer infants being fully vaccinated. Countries introducing new vaccines should (i) ensure adequate stock and resources for planned catch-up campaigns and strengthen system required for rapid roll-out and delivery. (ii) Understand who remains most vulnerable so that focussed delivery to improve access to immunisation occurs. This is crucial for maximising the equitable benefits of universal vaccination programmes. (iii) Ensure culturally appropriate and understandable health information about vaccines is widely available together with a continued focus on making vaccines as accessible as possible to families on the social margins. These suggested recommendations are essential if the full benefits of vaccination programmes are to be realized amongst the most vulnerable.

## Supporting Information

S1 Table Robust Poisson regression for factors associated with PCV13 or RV1 given at a later date than Pentavalent vaccine: “vaccine non-availability”. (DOCX)

S2 Table Univariable and multivariable analysis of predictors of pneumococcal vaccine uptake in the birth cohort. (DOCX)

S3 Table Univariable and multivariable analysis of predictors of rotavirus vaccine uptake. (DOCX)

S4 Table Univariable and multivariable analysis of predictors of measles vaccine uptake. (DOCX)

S5 Table Survival analysis of predictors of timeliness of pneumococcal vaccination. (DOCX)

S6 Table Survival analysis of predictors of timeliness of rotavirus vaccination. (DOCX)

S7 Table Survival analysis of predictors of timeliness of measles vaccination. (DOCX)

S8 Table Literature reporting risk factors for low vaccine uptake and/or late vaccination in sub-Saharan countries. (DOCX)

## References

[1] O'Hare B, Makuta I, Bar-Zeev N, Chiwaula L, Cobham A. The effect of illicit financial flows on time to reach the fourth Millennium Development Goal in Sub-Saharan Africa: a quantitative analysis. J R Soc Med. 2014;107(4):148–56. 10.1177/0141076813514575 24334911 PMC4109330

[2] Government of Malawi. Comprehensive EPI Multi-Year Plan 2012–2016. Accessed on 08/07/2015 at http://www.nationalplanningcycles.org/sites/default/files/country_docs/Malawi/document_no.pdf. 2011.

[3] WHO, UNICEF, World Bank. State of the world’s vaccines and immunization, 3rd ed Geneva, World Health Organization, 2009 Accessed on 11/05/2015 at http://whqlibdoc.who.int/publications/2009/9789241563864_eng.pdf

[4] Gram L, Soremekun S, ten Asbroek A, Manu A, O'Leary M, Hill Z, et al Socio-economic determinants and inequities in coverage and timeliness of early childhood immunisation in rural Ghana. Trop Med Int Health. 2014;19(7):802–11. 10.1111/tmi.12324 24766425

[5] Fadnes LT, Nankabirwa V, Sommerfelt H, Tylleskar T, Tumwine JK, Engebretsen IM. Is vaccination coverage a good indicator of age-appropriate vaccination? A prospective study from Uganda. Vaccine. 2011;29(19):3564–70. 10.1016/j.vaccine.2011.02.093 21402043

[6] Jahn A, Floyd S, Mwinuka V, Mwafilaso J, Mwagomba D, Mkisi RE, et al Ascertainment of childhood vaccination histories in northern Malawi. Trop Med Int Health. 2008;13(1):129–38. 10.1111/j.1365-3156.2007.01982.x 18291011

[7] Bar-Zeev N, Kapanda L, King C, Beard J, Phiri T, Mvula H, et al Methods and challenges in measuring the impact of national pneumococcal and rotavirus vaccine introduction on morbidity and mortality in Malawi. Vaccine. 2015;33(23):2637–45. 10.1016/j.vaccine.2015.04.053 25917672 PMC4441035

[8] Crampin AC, Dube A, Mboma S, Price A, Chihana M, Jahn A, et al Profile: the Karonga Health and Demographic Surveillance System. Int J Epidemiol. 2012;41(3):676–85. 10.1093/ije/dys088 22729235 PMC3396313

[9] Jahn A, Branson K, Crampin AC, Fine PE, Glynn JR, McGrath N, et al Evaluation of a village-informant driven demographic surveillance system in Karonga, Northern Malawi. Demographic Res. 2007;16:217–48.

[10] Sandvik B. World Borders Dataset—thematicmapping.org. 2009. Available from: http://thematicmapping.org/downloads/world_borders.php.

[11] Huber P. The behavior of maximum likelihood estimates under non-standard conditions. Proc Fifth Berkeley Symp Math Statist and Prob. 1967:221–33.

[12] White H. A heteroskedasticity-consistent covariance matrix estimator and a direct test for heteroskedasticity. Econometrica. 1980;48:817–30.

[13] Knol MJ, Le Cessie S, Algra A, Vandenbroucke JP, Groenwold RH. Overestimation of risk ratios by odds ratios in trials and cohort studies: alternatives to logistic regression. Can Med Assoc J. 2012;184(8):895–9. 10.1503/cmaj.101715 22158397 PMC3348192

[14] Button KS, Ioannidis JP, Mokrysz C, Nosek BA, Flint J, Robinson ES, et al Power failure: why small sample size undermines the reliability of neuroscience. Nat Rev Neurosci. 2013;14(5):365–76. 10.1038/nrn3475 23571845

[15] Abadura SA, Lerebo WT, Kulkarni U, Mekonnen ZA. Individual and community level determinants of childhood full immunization in Ethiopia: a multilevel analysis. BMC Public Health. 2015;15(1):972 10.1186/s12889-015-2315-z 26415507 PMC4587824

[16] Babalola S. Determinants of the uptake of the full dose of diphtheria-pertussis-tetanus vaccines (DPT3) in Northern Nigeria: a multilevel analysis. Matern Child Health J. 2009;13(4):550–8. 10.1007/s10995-008-0386-5 18607704

[17] Babirye JN, Engebretsen IM, Makumbi F, Fadnes LT, Wamani H, Tylleskar T, et al Timeliness of childhood vaccinations in Kampala Uganda: a community-based cross-sectional study. PLoS One. 2012;7(4):e35432 10.1371/journal.pone.0035432 22539972 PMC3335141

[18] Bosch-Capblanch X, Banerjee K, Burton A. Unvaccinated children in years of increasing coverage: how many and who are they? Evidence from 96 low- and middle-income countries. Trop Med Int Health. 2012;17(6):697–710. 10.1111/j.1365-3156.2012.02989.x 22943300

[19] Canavan ME, Sipsma HL, Kassie GM, Bradley EH. Correlates of complete childhood vaccination in East African countries. PLoS One. 2014;9(4):e95709 10.1371/journal.pone.0095709 24752178 PMC3994083

[20] Fadnes LT, Jackson D, Engebretsen IM, Zembe W, Sanders D, Sommerfelt H, et al Vaccination coverage and timeliness in three South African areas: a prospective study. BMC Public Health. 2011;11:404 10.1186/1471-2458-11-404 21619642 PMC3126743

[21] Favin M, Steinglass R, Fields R, Banerjee K, Sawhney M. Why children are not vaccinated: a review of the grey literature. Int Health. 2012;4(4):229–38. 10.1016/j.inhe.2012.07.004 24029668

[22] Glatman-Freedman A, Nichols K. The effect of social determinants on immunization programs. Hum Vaccin Immunother. 2012;8(3):293–301. 10.4161/hv.19003 22327490

[23] Jani JV, De Schacht C, Jani IV, Bjune G. Risk factors for incomplete vaccination and missed opportunity for immunization in rural Mozambique. BMC Public Health. 2008;8:161 10.1186/1471-2458-8-161 18485194 PMC2405792

[24] Le Polain de Waroux O, Schellenberg JR, Manzi F, Mrisho M, Shirima K, Mshinda H, et al Timeliness and completeness of vaccination and risk factors for low and late vaccine uptake in young children living in rural southern Tanzania. Int Health. 2013;5(2):139–47. 10.1093/inthealth/iht006 24030114

[25] Munthali AC. Determinants of vaccination coverage in Malawi: evidence from the demographic and health surveys. Malawi Med J. 2007;19(2):79–82. 23878640 10.4314/mmj.v19i2.10934 PMC3345647

[26] National Statistical Office (NSO) and ICF Macro. Malawi Demographic and Health Survey 2010. Zomba, Malawi, and Calverton, Maryland, USA: NSO and ICF Macro 2011.

[27] Odutola A, Afolabi MO, Ogundare EO, Lowe-Jallow YN, Worwui A, Okebe J, et al Risk factors for delay in age-appropriate vaccinations among Gambian children. BMC Health Serv Res. 2015;15:346 10.1186/s12913-015-1015-9 26315547 PMC4551385

[28] Payne S, Townend J, Jasseh M, Lowe Jallow Y, Kampmann B. Achieving comprehensive childhood immunization: an analysis of obstacles and opportunities in The Gambia. Health Policy Plan. 2014;29(2):193–203. 10.1093/heapol/czt004 23426974 PMC3944881

[29] Rainey JJ, Watkins M, Ryman TK, Sandhu P, Bo A, Banerjee K. Reasons related to non-vaccination and under-vaccination of children in low and middle income countries: findings from a systematic review of the published literature, 1999–2009. Vaccine. 2011;29(46):8215–21. 10.1016/j.vaccine.2011.08.096 21893149

[30] Schoeps A, Ouedraogo N, Kagone M, Sie A, Muller O, Becher H. Socio-demographic determinants of timely adherence to BCG, Penta3, measles, and complete vaccination schedule in Burkina Faso. Vaccine. 2013;32(1):96–102. 10.1016/j.vaccine.2013.10.063 . 24183978

[31] Wiysonge CS, Uthman OA, Ndumbe PM, Hussey GD. Individual and contextual factors associated with low childhood immunisation coverage in sub-Saharan Africa: a multilevel analysis. PLoS One. 2012;7(5):e37905 10.1371/journal.pone.0037905 22662247 PMC3360654

[32] Bar-Zeev N, Kapanda L, Tate JE, Jere KC, Iturriza-Gomara M, Nakagomi O, et al Effectiveness of a monovalent rotavirus vaccine in infants in Malawi after programmatic roll-out: an observational and case-control study. Lancet Infect Dis. 2015;15(4):422–8. 10.1016/s1473-3099(14)71060-6 25638521 PMC4374102

[33] Clinton Health Access Initiative. Annual Report 2013. p. 1–24. Accessed on 17 March 2015 at www.clintonhealthaccess.org/files/CHAI_Annual_Report_3.pdf.

[34] Scott JA, Ojal J, Ashton L, Muhoro A, Burbidge P, Goldblatt D. Pneumococcal conjugate vaccine given shortly after birth stimulates effective antibody concentrations and primes immunological memory for sustained infant protection. Clin Infect Dis. 2011;53(7):663–70. 10.1093/cid/cir444 21865175 PMC3166350

[35] Spijkerman J, Veenhoven RH, Wijmenga-Monsuur AJ, Elberse KE, van Gageldonk PG, Knol MJ, et al Immunogenicity of 13-valent pneumococcal conjugate vaccine administered according to 4 different primary immunization schedules in infants: a randomized clinical trial. JAMA. 2013;310(9):930–7. 10.1001/jama.2013.228052 24002279

[36] Heinsbroek E, Tafatatha T, Chisambo C, Phiri A, Mwiba O, Ngwira B, et al Pneumococcal Acquisition Among Infants Exposed to HIV in Rural Malawi: A Longitudinal Household Study. Am J Epidemiol. 2016;183(1):70–8. 10.1093/aje/kwv134 26628514 PMC4690474

