# Supplementary material for: Predictors of Uptake and Timeliness of Newly Introduced Pneumococcal and Rotavirus Vaccines, and of Measles Vaccine in Rural Malawi: A Population Cohort Study
Source: PLoS One. 2016 May 6;11(5):e0154997. doi: 10.1371/journal.pone.0154997 (PMC4859501; doi:10.1371/journal.pone.0154997)
Supplement: S1 Table — (DOCX) [file pone.0154997.s001.docx]

| **S1 Table. Robust Poisson regression for factors associated with PCV13 or RV1 given at a later date than Pentavalent vaccine: “vaccine non-availability”** | | | | | | | | |
| --- | --- | --- | --- | --- | --- | --- | --- | --- |
| **PCV13** | | | | | | | | |
| Variable |  | PCV13 dose 1 | | |  | PCV13 dose 3 | | |
|  | N | N (%) received dose later than Pentavalent | RR (95% CI) | aRR^1^ (95% CI) | N | N (%) received dose later than Pentavalent | RR (95% CI) | aRR^2^ (95% CI) |
| Time since vaccine introduction^3^ |  |  |  |  |  |  |  |  |
| 0-3 months | 214 | 47 (22.0%) | 1 | 1 | 190 | 78 (41.1%) | 1 | 1 |
| 4-6 months | 186 | 32 (17.2%) | 0.78 (0.52-1.17) | 0.97 (0.63-1.49) | 168 | 65 (38.7%) | 0.94 (0.73-1.22) | 0.94 (0.73-1.22) |
| 7-9 months | 170 | 27 (15.9%) | 0.72 (0.47-1.11) | 1.15 (0.65-2.04) | 163 | 54 (33.1%) | 0.81 (0.61-1.06) | 0.81 (0.61-1.06) |
| > 9 months | 517 | 57 (11.0%) | 0.50 (0.35-0.71) | 0.61 (0.41-0.90) | 486 | 79 (16.3%) | 0.40 (0.30-0.52) | 0.40 (0.30-0.52) |
| Distance to road (km) |  |  |  |  |  |  |  |  |
| <1 | 823 | 119 (14.5%) | 1 | 1 | 766 | 202 (26.4%) | 1 | 1 |
| 1-1.49 | 146 | 19 (13.0%) | 0.90 (0.57-1.41) | 0.89 (0.57-1.41) | 138 | 48 (34.8%) | 1.31 (1.02-1.71) | 1.29 (1.00-1.66) |
| ≥ 1.5 | 118 | 25 (21.2%) | 1.47 (1.00-2.15) | 1.57 (1.07-2.30) | 103 | 26 (25.2%) | 0.96 (0.67-1.36) | 1.13 (0.80-1.59) |
| Season^4^ |  |  |  |  |  |  |  |  |
| Dry | 566 | 71 (12.5%) | 1 | 1 | 600 | 174 (29.0%) | 1 | 1 |
| Rainy | 521 | 92 (17.7%) | 1.60 (1.20-2.14) | 1.63 (1.12-2.37) | 407 | 102 (25.1%) | 0.86 (0.70-1.07) | 0.97 (0.75-1.25) |

^1^Adjusted for distance to the main road, season and time since vaccine introduction

^2^Adjusted for time since vaccine introduction

^3^Time between vaccine introduction and PCV13 due date

^4^ Season at time of Pentavalent receipt: dry season = May-November, rainy season = December-April

| **RV1** | | | | | | | | |
| --- | --- | --- | --- | --- | --- | --- | --- | --- |
|  |  | RV1 dose 1 | | |  |  | RV1 dose 2 |  |
| Variable | N | N (%) received dose later than Pentavalent | RR (95% CI) | aRR^1^ (95% CI) | N | N (%) received dose later than Pentavalent | RR (95% CI) | aRR^2^ (95% CI) |
| Time since vaccine introduction^3^ |  |  |  |  |  |  |  |  |
| 0-3 months | 79 | 18 (22.8%) | 1 | 1 | 77 | 30 (39.0%) | 1 | 1 |
| 4-6 months | 90 | 23 (25.6%) | 1.58 (0.97-2.56) | 1.64 (1.02-2.64) | 87 | 33 (37.9%) | 1.11 (0.79-1.55) | 1.14 (0.82-1.58) |
| > 6 months | 179 | 29 (16.2%) | 1.41 (0.83-2.38) | 1.55 (0.91-2.64) | 172 | 59 (34.3%) | 1.14 (0.80-1.61) | 1.27 (0.87-1.84) |
| Distance to road (km) |  |  |  |  |  |  |  |  |
| <1 | 270 | 44 (16.3%) | 1 | 1 | 265 | 86 (32.5%) | 1 | 1 |
| 1-1.49 | 37 | 16 (43.2%) | 2.65 (1.68-4.19) | 2.65 (1.68-4.19) | 34 | 23 (67.7%) | 2.08 (1.56-2.79) | 2.08 (1.56-2.79) |
| ≥ 1.5 | 41 | 10 (24.4%) | 1.50 (0.82-2.74) | 1.50 (0.82-2.74) | 37 | 13 (35.1%) | 1.08 (0.68-1.73) | 1.08 (0.68-1.73) |
| Season^4^ |  |  |  |  |  |  |  |  |
| Dry | 154 | 34 (22.1%) | 1 | 1 | 137 | 48 (35.0%) | 1 | 1 |
| Rainy | 194 | 36 (18.6%) | 0.84 (0.55-1.28) | 0.86 (0.57-1.30) | 199 | 74 (37.2%) | 1.06 (0.79-1.42) | 1.02 (0.76-1.37) |
|  | | | | | | | | |

^1^ Adjusted for distance to the main road and time since vaccine introduction

^2^ Adjusted for distance to the main road

^3^ Time between vaccine introduction and RV1 due date

^4^ Season at time of Pentavalent receipt: dry season = May-November, rainy season = December-April
