# Supplementary material for: Predictors of Uptake and Timeliness of Newly Introduced Pneumococcal and Rotavirus Vaccines, and of Measles Vaccine in Rural Malawi: A Population Cohort Study
Source: PLoS One. 2016 May 6;11(5):e0154997. doi: 10.1371/journal.pone.0154997 (PMC4859501; doi:10.1371/journal.pone.0154997)
Supplement: S2 Table — (DOCX) [file pone.0154997.s002.docx]

| **S2 Table. Univariable and multivariable analysis of predictors of pneumococcal vaccine uptake in the birth cohort** | | | | | | | | |
| --- | --- | --- | --- | --- | --- | --- | --- | --- |
| Variable | N | Predictors of being vaccinated with one dose of PCV13 | | | N | Predictors of being vaccinated with three doses of PCV13 | | |
|  |  | Coverage^1^ (%) | Crude RR (95% CI) | Adjusted RR^2^ (95% CI) |  | Coverage (%) | Crude RR (95% CI) | Adjusted RR^3^ (95% CI) |
| Gender |  |  |  |  |  |  |  |  |
| Female | 634 | 95.1 | 1 | 1 | 632 | 89.7 | 1 | 1 |
| Male | 583 | 95.7 | 1.01 (0.98- 1.03) | 1.00 (0.98- 1.03) | 583 | 89.0 | 1.00 (0.96- 1.03) | 0.99 (0.95- 1.03) |
| First dose PCV13 given later than Pentavalent |  |  |  |  |  |  |  |  |
| No | - | - | - | - | 922 | 96.2 | 1 | 1 |
| Yes | - | - | - | - | 163 | 79.1 | 0.82 (0.76- 0.89) | 0.82 (0.76- 0.89) |
| Second dose PCV13 given later than Pentavalent |  |  |  |  |  |  |  |  |
| No | - | - | - | - | 834 | 97.6 | 1 | 1 |
| Yes | - | - | - | - | 243 | 84.0 | 0.86 (0.81- 0.91) | 0.86 (0.81- 0.91) |
| Time since vaccine introduction^4^ |  |  |  |  |  |  |  |  |
| 0-3 months | 268 | 90.7 | 1 | 1 | 268 | 81.7 | 1 | 1 |
| 4-6 months | 220 | 92.7 | 1.02 (0.97- 1.08) | 1.03 (0.97- 1.08) | 220 | 86.4 | 1.06 (0.98- 1.14) | 1.07 (0.99- 1.15) |
| 7-9 months | 194 | 94.3 | 1.04 (0.99- 1.10) | 1.02 (0.97- 1.07) | 194 | 89.7 | 1.10 (1.02- 1.18) | 1.10 (1.02- 1.18) |
| > 9 months | 535 | 99.3 | 1.09 (1.05- 1.14) | 1.09 (1.05- 1.13) | 533 | 94.4 | 1.15 (1.09- 1.23) | 1.16 (1.09- 1.23) |
| Mother’s age |  |  |  |  |  |  |  |  |
| <20 | 205 | 94.2 | 1 | 1 | 205 | 88.8 | 1 | 1 |
| 20-29 | 662 | 94.7 | 1.10 (0.97- 1.05) | 0.99 (0.95- 1.03) | 661 | 88.5 | 1.00 (0.94- 1.05) | 0.99 (0.94- 1.05) |
| 30-39 | 312 | 97.4 | 1.03 (1.00- 1.08) | 1.02 (0.98- 1.06) | 312 | 91.0 | 1.03 (0.97- 1.09) | 1.03 (0.97- 1.09) |
| ≥ 40 | 36 | 97.2 | 1.03 (0.97- 1.10) | 1.01 (0.95- 1.08) | 35 | 94.3 | 1.06 (0.97- 1.17) | 1.07 (0.98- 1.17) |
| Mother’s education |  |  |  |  |  |  |  |  |
| <5 years primary | 77 | 96.1 | 1 | 1 | 77 | 85.7 | 1 | 1 |
| >= 5 years primary | 791 | 95.5 | 0.99 (0.95- 1.04) | 0.99 (0.95- 1.05) | 790 | 89.0 | 1.04 (0.94- 1.14) | 1.04 (0.95- 1.14) |
| Secondary / tertiary | 348 | 95.1 | 0.99 (0.94- 1.04) | 0.99 (0.94- 1.04) | 347 | 91.1 | 1.06 (0.96- 1.17) | 1.05 (0.95- 1.16) |
| Mother’s marital status |  |  |  |  |  |  |  |  |
| Married | 1085 | 95.6 | 1 | 1 | 1083 | 89.6 | 1 | 1 |
| Unmarried^5^ | 130 | 93.9 | 0.98 (0.94- 1.03) | 0.97 (0.93- 1.02) | 130 | 87.7 | 0.98 (0.92- 1.05) | 0.97 (0.90- 1.03) |
| Mother mobile phone personal use |  |  |  |  |  |  |  |  |
| No | 926 | 96.1 | 1 | 1 | 962 | 90.1 | 1 | 1 |
| Yes | 155 | 93.9 | 0.98 (0.94- 1.02) | 0.96 (0.92- 1.00) | 165 | 90.9 | 1.01 (0.96- 1.06) | 0.98 (0.93- 1.04) |
| Mother’s occupation |  |  |  |  |  |  |  |  |
| Farming | 1113 | 95.2 | 1 | 1 | 1111 | 88.8 | 1 | 1 |
| Other | 82 | 98.8 | 1.04 (1.01- 1.07) | 1.04 (1.02- 1.07) | 82 | 95.1 | 1.07 (1.02- 1.13) | 1.07 (1.02- 1.13) |
| Orphanhood |  |  |  |  |  |  |  |  |
| Both parents alive | 1194 | 95.3 | 1 | 1 | 1192 | 89.3 | 1 | 1 |
| Father died | 11 | 100 | 1.05 (1.04- 1.06) | 1.05 (1.02- 1.08) | 11 | 90.9 | 1.02 (0.15- 9.47) | 1.02 (0.85- 1.21) |
| Mother died | 5 | 100 | 1.05 (1.04- 1.06 | 1.01 (0.98- 1.05) | 5 | 100 | 1.12 (1.09- 1.14) | 1.08 (1.03- 1.13) |
| Place of birth |  |  |  |  |  |  |  |  |
| Health centre | 1091 | 95.4 | 1 | 1 | 1089 | 90.1 | 1 | 1 |
| Home / TBA / other | 114 | 94.7 | 0.99 (0.95- 1.04) | 1.00 (0.96- 1.05) | 114 | 83.3 | 0.93 (0.85- 1.01) | 0.94 (0.87- 1.03) |
| Housing standard |  |  |  |  |  |  |  |  |
| 1 (lowest) | 158 | 93.0 | 1 | 1 | 157 | 82.8 | 1 | 1 |
| 2 | 401 | 95.5 | 1.03 (0.98- 1.08) | 1.02 (0.97- 1.07) | 400 | 90.0 | 1.09 (1.00- 1.18) | 1.06 (0.98- 1.14) |
| 3 | 168 | 96.4 | 1.04 (0.98- 1.09) | 1.03 (0.97- 1.08) | 168 | 88.7 | 1.07 (0.98- 1.17) | 1.03 (0.95- 1.13) |
| 4 (highest) | 148 | 93.9 | 1.01 (0.95- 1.07) | 1.00 (0.94- 1.06) | 148 | 89.9 | 1.09 (0.99- 1.19) | 1.04 (0.95- 1.13) |
| Household size (persons) |  |  |  |  |  |  |  |  |
| <4 | 256 | 92.6 | 1 | 1 | 256 | 85.9 | 1 | 1 |
| 4-6 | 641 | 96.1 | 1.04 (1.00- 1.08) | 1.04 (1.00- 1.08) | 640 | 90.9 | 1.06 (1.00- 1.12) | 1.05 (1.00- 1.11) |
| ≥ 7 | 320 | 96.3 | 1.04 (1.00- 1.08) | 1.04 (1.00- 1.09) | 319 | 89.0 | 1.04 (0.97- 1.10) | 1.04 (0.98- 1.10) |
| Number of children <5 years in household |  |  |  |  |  |  |  |  |
| 1 | 495 | 94.1 | 1 | 1 | 495 | 88.3 | 1 | 1 |
| 2 | 621 | 96.0 | 1.02 (0.99- 1.05) | 1.00 (0.97- 1.03) | 619 | 89.8 | 1.02 (0.98- 1.06) | 1.02 (0.98- 1.07) |
| ≥ 3 | 101 | 98.0 | 1.04 (1.00- 1.08) | 1.03 (0.99- 1.08) | 101 | 92.1 | 1.04 (0.98- 1.11) | 1.07 (1.00- 1.13) |
| Distance to road (km) |  |  |  |  |  |  |  |  |
| <1 | 922 | 95.3 | 1 | 1 | 921 | 89.8 | 1 | 1 |
| 1-1.49 | 164 | 95.1 | 1.00 (0.96- 1.04) | 1.00 (0.96- 1.04) | 163 | 92.0 | 1.02 (0.97- 1.08) | 1.05 (1.00- 1.10) |
| ≥ 1.5 | 131 | 96.2 | 1.00 (1.05- 1.05) | 1.00 (0.96- 1.04) | 131 | 83.2 | 0.93 (0.86- 1.00) | 0.95 (0.87- 1.03) |
| Distance to clinic (km) |  |  |  |  |  |  |  |  |
| <1 | 856 | 96.1 | 1 | 1 | 854 | 90.8 | 1 | 1 |
| 1-1.49 | 249 | 94.8 | 0.99 (0.95- 1.02) | 0.98 (0.95- 1.02) | 249 | 88.8 | 0.98 (0.93- 1.03) | 0.98 (0.93- 1.03) |
| ≥ 1.5 | 112 | 91.1 | 0.95 (0.89- 1.01) | 0.95 (0.90- 1.01) | 112 | 80.4 | 0.86 (0.81- 0.97) | 0.89 (0.81- 0.98) |
| Moved house |  |  |  |  |  |  |  |  |
| No | 1155 | 95.7 | 1 | 1 | 1153 | 89.9 | 1 | 1 |
| Yes | 62 | 90.3 | 0.94 (0.87- 1.03) | 0.96 (0.88- 1.04) | 62 | 80.7 | 0.90 (0.79- 1.02) | 0.91 (0.80- 1.03) |
| Season^6^ |  |  |  |  |  |  |  |  |
| Dry | 621 | 97.1 | 1 | 1 | 619 | 92.7 | 1 | 1 |
| Rainy | 596 | 93.6 | 0.96 (0.94- 0.99) | 0.97 (0.95- 1.00) | 596 | 85.9 | 0.97 (0.93- 1.00) | 0.97 (0.93- 1.01) |

PCV = Pneumococcal Conjugate Vaccine, TBA = Traditional Birth Attendant

^1^ Coverage is percent vaccinated

^2^ Adjusted for age at onset of vaccination, number of household members, season and maternal occupation

^3^ Adjusted for age at onset of vaccination, distance to the nearest clinic and maternal occupation

^4^ Time between vaccine introduction and due date of first dose PCV

^5^ Never married/divorced/widowed

^6^ Season at due date of PCV: dry season = May-November, rainy season = December-April
