# Supplementary material for: Predictors of Uptake and Timeliness of Newly Introduced Pneumococcal and Rotavirus Vaccines, and of Measles Vaccine in Rural Malawi: A Population Cohort Study
Source: PLoS One. 2016 May 6;11(5):e0154997. doi: 10.1371/journal.pone.0154997 (PMC4859501; doi:10.1371/journal.pone.0154997)
Supplement: S3 Table — (DOCX) [file pone.0154997.s003.docx]

| **S3 Table. Univariable and multivariable analysis of predictors of rotavirus vaccine uptake** | | | | | | | | |
| --- | --- | --- | --- | --- | --- | --- | --- | --- |
| Variable | N | Predictors of being vaccinated with one dose of RV1 | | | N | Predictors of being vaccinated with two doses of RV1 | | |
|  |  | Coverage^1^ (%) | Crude RR (95% CI) | Adjusted RR^2^ (95% CI) |  | Coverage (%) | Crude RR (95% CI) | Adjusted RR^3^ (95% CI) |
| Gender |  |  |  |  |  |  |  |  |
| Female | 201 | 90.1 | 1 | 1 | 201 | 86.1 | 1 | 1 |
| Male | 196 | 90.3 | 1.00 (0.94- 1.07) | 1.01 (0.50- 2.04) | 195 | 87.7 | 1.02 (0.94- 1.10) | 1.01 (0.94- 1.09) |
| First dose RV1 given later than Pentavalent |  |  |  |  |  |  |  |  |
| No | - | - | - | - | 278 | 96.4 | 1 | 1 |
| Yes | - | - | - | - | 69 | 94.3 | 0.99 (0.94- 1.05) | 1.00 (0.95- 1.05) |
| Time since vaccine introduction^4^ |  |  |  |  |  |  |  |  |
| 0-3 months | 198 | 91.9 | 1 | 1 | 197 | 88.3 | 1 | 1 |
| 4-6 months | 109 | 83.5 | 0.91 (0.83- 1.00) | 0.92 (0.84- 1.00) | 109 | 80.7 | 0.91 (0.82- 1.02) | 0.93 (0.84- 1.02) |
| >7 months | 90 | 94.4 | 1.03 (0.96- 1.10) | 1.03 (0.96- 1.10) | 90 | 91.1 | 1.03 (0.95- 1.12) | 1.03 (0.94- 1.13) |
| Mother’s age (years) |  |  |  |  |  |  |  |  |
| <20 | 62 | 93.6 | 1 | 1 | 62 | 90.3 | 1 | 1 |
| 20-29 | 228 | 89.5 | 0.96 (0.88- 1.04) | 1.00 (0.92- 1.07) | 227 | 86.3 | 0.96 (0.87- 1,05) | 1.00 (0.91- 1.09) |
| 30-39 | 98 | 88.8 | 0.95 (0.86- 1.04) | 0.98 (0.90- 1.07) | 98 | 84.7 | 0.94 (0.83- 1.05) | 0.97 (0.87- 1.08) |
| ≥ 40 | 8 | 100 | 1.07 (1.00- 1.14) | 1.07 (0.99- 1.16) | 8 | 100 | 1.11 (1.02- 1.20) | 1.10 (1.00- 1.21) |
| Mother’s education |  |  |  |  |  |  |  |  |
| <5 years primary | 27 | 74.1 | 1 | 1 | 27 | 70.4 | 1 | 1 |
| ≥ 5 years primary | 260 | 90.8 | 1.23 (0.98- 1.54) | 1.19 (0.97- 1.45) | 259 | 86.9 | 1.23 (0.96- 1.58) | 1.19 (0.95- 1.49) |
| Secondary / tertiary | 109 | 92.7 | 1.25 (0.99- 1.57) | 1.19 (0.97- 1.46) | 109 | 90.8 | 1.29 (1.00- 1.66) | 1.22 (0.97- 1.53) |
| Mother’s marital status |  |  |  |  |  |  |  |  |
| Married | 354 | 89.6 | 1 | 1 | 353 | 86.4 | 1 | 1 |
| Unmarried^5^ | 42 | 95.2 | 1.06 (0.99- 1.15) | 1.08 (1.01- 1.15) | 42 | 90.5 | 1.05 (0.94- 1.16) | 1.05 (0.96- 1.15) |
| Mother mobile phone |  |  |  |  |  |  |  |  |
| No | 326 | 89.6 | 1 | 1 | 325 | 86.2 | 1 | 1 |
| Yes | 65 | 92.3 | 1.03 (0.95- 1.12) | 1.02 (0.94- 1.10) | 65 | 90.8 | 1.05 (0.96- 1.15) | 1.04 (0.95- 1.13) |
| Mother’s occupation |  |  |  |  |  |  |  |  |
| Farming | 365 | 90.1 | 1 | 1 | 364 | 86.8 | 1 | 1 |
| Other | 27 | 88.9 | 0.99 (0.85- 1.13) | 0.96 (0.86- 1.07) | 27 | 88.9 | 1.02 (0.89- 1.18) | 0.98 (0.88- 1.10) |
| Orphanhood |  |  |  |  |  |  |  |  |
| Both parents alive | 389 | 90.0 | 1 | 1 | 388 | 86.7 | 1 | 1 |
| Father died | 4 | 100 | 1.11 (1.08- 1.15) | 1.22 (1.02- 1.46) | 4 | 75.0 | 0.86 (0.49- 1.52) | 0.96 (0.64- 1.45) |
| Mother died | 2 | 100 | 1.11 (1.08- 1.15) | 1.12 (0.91- 1.38) | 2 | 100 | 1.15 (1.11- 1.20) | 1.20 (0.96- 1.49) |
| Place of birth |  |  |  |  |  |  |  |  |
| Health centre | 367 | 90.2 | 1 | 1 | 366 | 87.4 | 1 | 1 |
| Home / TBA / other | 25 | 88.0 | 0.98 (0.84- 1.13) | 0.97 (0.91- 1.03) | 25 | 76.0 | 0.87 (0.69- 1.09) | 0.93 (0.74- 1.15) |
| Housing standard |  |  |  |  |  |  |  |  |
| 1 (lowest) | 42 | 86.7 | 1 | 1 | 41 | 82.9 | 1 | 1 |
| 2 | 127 | 88.2 | 1.03 (0.90- 1.18) | 0.95 (0.81- 1.10) | 127 | 84.3 | 1.02 (0.87- 1.19) | 0.93 (0.77- 1.11) |
| 3 | 54 | 87.0 | 1.02 (0.86- 1.19) | 0.95 (0.81- 1.12) | 54 | 85.2 | 1.03 (0.86- 1.23) | 0.95 (0.79- 1.14) |
| 4 (highest) | 38 | 94.7 | 1.11 (0.96- 1.28) | 0.97 (0.83- 1.14) | 38 | 89.5 | 1.08 (0.90- 1.29) | 0.93 (0.76- 1.13) |
| Household size (persons) |  |  |  |  |  |  |  |  |
| <4 | 70 | 92.9 | 1 | 1 | 70 | 90.0 | 1 | 1 |
| 4-6 | 220 | 88.6 | 0.95 (0.88- 1.03) | 1.00 (0.91- 1.09) | 219 | 85.8 | 0.95 (0.87- 1.04) | 0.98 (0.89- 1.09) |
| ≥ 7 | 107 | 91.6 | 0.99 (0.90- 1.08) | 1.06 (0.97- 1.16) | 107 | 86.9 | 0.97 (0.87- 1.08) | 1.02 (0.92- 1.14) |
| Number of children <5 years in household |  |  |  |  |  |  |  |  |
| 1 | 148 | 94.6 | 1 | 1 | 148 | 91.9 | 1 | 1 |
| 2 | 226 | 88.9 | 0.94 (0.89- 1.00) | 0.97 (0.91- 1.03) | 225 | 85.3 | 0.93 (0.86- 1.00) | 0.96 (0.90- 1.03) |
| ≥ 3 | 23 | 73.9 | 0.78 (0.61- 1.00) | 0.81 (0.63- 1.03) | 23 | 69.6 | 0.76 (0.58- 1.00) | 0.77 (0.59- 1.00) |
| Distance to road (km) |  |  |  |  |  |  |  |  |
| <1 | 297 | 93.9 | 1 | 1 | 297 | 91.6 | 1 | 1 |
| 1-1.49 | 45 | 84.4 | 0.90 (0.79- 1.02) | 0.90 (0.80- 1.02) | 45 | 77.8 | 0.85 (0.72- 1.00) | 0.85 (0.73- 1.00) |
| ≥ 1.5 | 55 | 74.6 | 0.79 (0.68- 0.93) | 0.80 (0.69- 0.93) | 54 | 68.5 | 0.75 (0.62- 0.90) | 0.75 (0.63- 0.90) |
| Distance to clinic (km) |  |  |  |  |  |  |  |  |
| <1 | 284 | 92.3 | 1 | 1 | 283 | 89.1 | 1 | 1 |
| 1-1.49 | 84 | 88.1 | 0.95 (0.88- 1.04) | 0.98 (0.91- 1.07) | 84 | 85.7 | 0.96 (0.87- 1.06) | 1.00 (0.91- 1.10) |
| ≥ 1.5 | 29 | 75.9 | 0.82 (0.67- 1.01) | 0.90 (0.73- 1.11) | 29 | 69.0 | 0.77 (0.60- 0.99) | 0.85 (0.65- 1.10) |
| Moved house |  |  |  |  |  |  |  |  |
| No | 383 | 90.1 | 1 | 1 | 382 | 87.2 | 1 | 1 |
| Yes | 14 | 92.9 | 1.03 (0.89- 1.20) | 1.07 (0.90- 1.28) | 14 | 78.6 | 0.90 (0.68- 1.19) | 0.96 (0.74- 1.24) |
| Season^6^ |  |  |  |  |  |  |  |  |
| Dry | 178 | 91.6 | 1 | 1 | 178 | 89.3 | 1 | 1 |
| Rainy | 219 | 89.0 | 0.97 (0.88- 0.96) | 1.01 (0.84- 1.10) | 218 | 84.9 | 0.95 (0.88- 1.03) | 0.95 (0.88- 1.03) |
| RV1 = Monovalent Rotavirus Vaccine, TBA = Traditional Birth Attendant  ^1^ Coverage is percent vaccinated  ^2^ Adjusted for age at onset of vaccination, distance to the nearest main road and number of children <5 years in the household  ^2^ Adjusted for distance to the nearest main road and number of children <5 years in the household  ^4^ Time between vaccine introduction and due date of first dose RV1  ^5^ Never married/divorced/widowed  ^6^ Season at due date of RV1 receipt: dry season = May-November, rainy season = December-April | | | | | | | | |
