# Supplementary material for: Predictors of Uptake and Timeliness of Newly Introduced Pneumococcal and Rotavirus Vaccines, and of Measles Vaccine in Rural Malawi: A Population Cohort Study
Source: PLoS One. 2016 May 6;11(5):e0154997. doi: 10.1371/journal.pone.0154997 (PMC4859501; doi:10.1371/journal.pone.0154997)
Supplement: S4 Table — (DOCX) [file pone.0154997.s004.docx]

| **S4 Table. Univariable and multivariable analysis of predictors of measles vaccine uptake** | | | | | |
| --- | --- | --- | --- | --- | --- |
| Variable | N | Coverage (%) | RR (95% CI) | aRR^1^ (95% CI) | |
| Gender |  |  |  | |  |
| Female | 1025 | 87.5 | 1 | | 1 |
| Male | 995 | 87.9 | 1.00 (0.97-1.04) | | 1.00 (0.97-1.04) |
| Mother’s age (yrs) |  |  |  | |  |
| <20 | 332 | 91.0 | 1 | | 1 |
| 20-29 | 1093 | 88.0 | 0.97 (0.93-1.01) | | 0.99 (0.95-1.03) |
| 30-39 | 530 | 85.9 | 0.94 (0.90-0.99) | | 0.96 (0.92-1.01) |
| ≥ 40 | 63 | 82.5 | 0.91 (0.81-1.02) | | 0.91 (0.81-1.03) |
| Mother’s education |  |  |  | |  |
| <5 years primary | 146 | 80.8 | 1 | | 1 |
| >= 5 years primary | 1304 | 87.4 | 1.08 (0.97-1.17) | | 1.08 (0.99-1.17) |
| Secondary / tertiary | 568 | 90.1 | 1.12 (1.03-1.21) | | 1.09 (1.00-1.18) |
| Mother’s marital status |  |  |  | |  |
| Married | 1801 | 88.0 | 1 | | 1 |
| Unmarried^2^ | 215 | 85.6 | 0.97 (0.92-1.03) | | 0.95 (0.89-1.00) |
| Mother mobile phone |  |  |  | |  |
| No | 1478 | 87.1 | 1 | | 1 |
| Yes | 245 | 88.2 | 1.01 (0.96-1.06) | | 0.99 (0.95-1.05) |
| Mother’s occupation |  |  |  | |  |
| Farming | 1744 | 87.1 | 1 | | 1 |
| Other | 130 | 96.2 | 1.10 (1.06-1.15) | | 1.09 (1.05-1.13) |
| Orphanhood |  |  |  | |  |
| Both parents alive | 1976 | 87.6 | 1 | | 1 |
| Father died | 23 | 87.0 | 0.99 (0.85-1.16) | | 1.00 (0.86-1.16) |
| Mother died | 7 | 100.0 | 1.14 (1.12-1.16) | | 1.12 (1.09-1.16) |
| Place of birth |  |  |  | |  |
| Health centre | 1787 | 88.3 | 1 | | 1 |
| Home / TBA / other | 191 | 81.7 | 0.93 (0.86-0.99) | | 0.94 (0.88-1.01) |
| Housing standard |  |  |  | |  |
| 1 (lowest) | 293 | 84.0 | 1 | | 1 |
| 2 | 694 | 86.7 | 1.03 (0.98-1.09) | | 1.02 (0.96-1.09) |
| 3 | 325 | 88.3 | 1.05 (0.99-1.12) | | 1.03 (0.96-1.10) |
| 4 (highest) | 278 | 91.1 | 1.08 (1.02-1.15) | | 1.04 (0.97-1.11) |
| Household size (persons) |  |  |  | |  |
| <4 | 409 | 90.7 | 1 | | 1 |
| 4-6 | 1057 | 88.0 | 0.97 (0.93-1.00) | | 1.01 (0.97-1.06) |
| ≥ 7 | 554 | 85.0 | 0.94 (0.89-0.98) | | 0.98 (0.93-1.03) |
| Number of children <5y in household |  |  |  | |  |
| 1 | 837 | 91.2 | 1 | | 1 |
| 2 | 1035 | 84.8 | 0.93 (0.90-0.96) | | 0.94 (0.91-0.97) |
| ≥ 3 | 148 | 88.5 | 0.97 (0.91-1.03) | | 0.99 (0.93-1.05) |
| Distance to road (km) |  |  |  | |  |
| <1 | 1526 | 88.7 | 1 | | 1 |
| 1-1.49 | 295 | 85.8 | 0.97 (0.92-1.02) | | 1.00 (0.95-1.05) |
| ≥ 1.5 | 199 | 83.4 | 0.94 (0.88-1.00) | | 0.98 (0.92-1.05) |
| Distance to clinic (km) |  |  |  | |  |
| <1 | 1397 | 89.3 | 1 | | 1 |
| 1-1.49 | 431 | 85.9 | 0.96 (0.92-1.00) | | 0.97 (0.93-1.01) |
| ≥ 1.5 | 192 | 80.7 | 0.90 (0.84-0.97) | | 0.91 (0.85-0.98) |
| Moved house |  |  |  | |  |
| No | 1931 | 87.9 | 1 | | 1 |
| Yes | 89 | 84.3 | 0.96 (0.88-1.05) | | 0.96 (0.88-1.05) |
| Season^3^ |  |  |  | |  |
| Dry | 1158 | 87.9 | 1 | | 1 |
| Rainy | 862 | 87.5 | 1.00 (0.96- 1.03) | | 0.99 (0.96- 1.04) |
| ^1^ Adjusted for maternal occupation, children <5 years in household, distance to clinic  ^2^ Never married/divorced/widowed  ^3^ At time measles vaccine dose due | | | | | |

.
