# Supplementary material for: Predictors of Uptake and Timeliness of Newly Introduced Pneumococcal and Rotavirus Vaccines, and of Measles Vaccine in Rural Malawi: A Population Cohort Study
Source: PLoS One. 2016 May 6;11(5):e0154997. doi: 10.1371/journal.pone.0154997 (PMC4859501; doi:10.1371/journal.pone.0154997)
Supplement: S5 Table — (DOCX) [file pone.0154997.s005.docx]

| **S5 Table. Survival analysis of predictors of timeliness of pneumococcal vaccination** | | | | |
| --- | --- | --- | --- | --- |
| Variable | N | Median delay in days (IQR) | Crude hazard ratio (95%CI) | Adjusted hazard ratio^1^ (95%CI) |
| Gender |  |  |  |  |
| Female | 601 | 51 (25-102) | 1 | 1 |
| Male | 555 | 53 (23-110) | 0.97 (0.86-1.10) | 0.99 (0.87-1.13) |
| Mother’s age (yrs) |  |  |  |  |
| <20 | 186 | 54 (22-102) | 1 | 1 |
| 20-29 | 629 | 57 (25-114) | 0.97 (0.82-1.16) | 0.96 (0.80-1.15) |
| 30-39 | 304 | 46 (23-97) | 1.10 (0.90-1.33) | 1.09 (0.89-1.32) |
| ≥ 40 | 35 | 40 (21-99) | 1.27 (0.87-1.85) | 1.25 (0.85-1.84) |
| Mother’s education |  |  |  |  |
| <5 years primary | 74 | 72 (27-143) | - | - |
| >= 5 years primary | 751 | 57 (25-114) | 1.19 (0.92-1.54) | 1.15 (0.88-1.50) |
| Secondary / tertiary | 330 | 41 (19-92) | 1.43 (1.09-1.87) | 1.27 (0.96-1.68) |
| Mother’s marital status |  |  |  |  |
| Married | 1028 | 54 (24-107) | 1 | 1 |
| Unmarried ^2^ | 126 | 46 (19-119) | 1.00 (0.82-1.21) | 0.97 (0.79-1.19) |
| Mother mobile phone |  |  |  |  |
| No | 919 | 55 (24-105) | 1 | 1 |
| Yes | 159 | 39 (19-79) | 1.21 (1.01, 1.44) | 1.08 (0.90-1.30) |
| Mother’s occupation |  |  |  |  |
| Farming | 1056 | 55 (24-113) | 1 | 1 |
| Other | 80 | 32 (18-49) | 1.69 (1.33-2.14) | 1.80 (1.41-2.28) |
| Orphanhood |  |  |  |  |
| Both parents alive | 1133 | 51 (24-109) | 1 | 1 |
| Father died | 11 | 46 (13-113) | 1.03 (0.55-1.92) | 1.20 (0.64-2.24) |
| Mother died | 5 | 42 (7-69) | 1.66 (0.69-4.00) | 2.64 (0.98-7.12) |
| Place of birth |  |  |  |  |
| Health centre | 1040 | 49 (23-100) | 1 | 1 |
| Home / TBA / other | 104 | 71 (35-193) | 0.71 (0.57-0.88) | 0.77 (0.61-0.97) |
| Housing standard |  |  |  |  |
| 1 (lowest) | 146 | 77 (34-166) | 1 | 1 |
| 2 | 380 | 53 (24-112) | 1.35 (1.09-1.66) | 1.25 (1.00-1.55) |
| 3 | 160 | 55 (24-107) | 1.34 (1.05-1.71) | 1.22 (0.95-1.57) |
| 4 (highest) | 146 | 41 (79-91) | 1.58 (1.23-2.02) | 1.27 (0.97-1.66) |
| Household size (persons) |  |  |  |  |
| <4 | 235 | 52 (23-110) | 1 | 1 |
| 4-6 | 611 | 50 (23-101) | 1.10 (0.94-1.30) | 1.10 (0.93-1.29) |
| ≥ 7 | 310 | 56 (25-115) | 1.01 (0.85-1.22) | 1.01 (0.84-1.21) |
| Number of children <5 years in household |  |  |  |  |
| 1 | 469 | 46 (21-93) | 1 | 1 |
| 2 | 593 | 58 (26-117) | 0.91 (0.80-1.03) | 0.95 (0.83-1.08) |
| ≥ 3 | 94 | 48 (25-102) | 1.01 (0.80-1.28) | 1.19 (0.93-1.50) |
| Distance to road (km) |  |  |  |  |
| <1 | 875 | 46 (22-98) | 1 | 1 |
| 1-1.49 | 155 | 56 (31-92) | 0.96 (0.81-1.15) | 1.02 (0.85-1.22) |
| ≥ 1.5 | 126 | 86 (47-156) | 0.65 (0.53-0.79) | 0.68 (0.55-0.84) |
| Distance to clinic (km) |  |  |  |  |
| <1 | 820 | 48 (22-99) | 1 | 1 |
| 1-1.49 | 230 | 63 (27-111) | 0.89 (0.76-1.04) | 0.91 (0.78-1.07) |
| ≥ 1.5 | 106 | 57 (30-160) | 0.71 (0.57-0.89) | 0.80 (0.63-1.01) |
| Moved house |  |  |  |  |
| No | 1102 | 49 (23-101) | 1 | 1 |
| Yes | 54 | 96 (68-199) | 0.54 (0.40-0.74) | 0.50 (0.37-0.69) |
| Season^3^ |  |  |  |  |
| Dry | 606 | 50 (25-99) | 1 | 1 |
| Rainy | 550 | 55 (22-119) | 0.90 (0.79- 1.02) | 0.86 (0.76- 0.98) |
| TBA = Traditional Birth Attendant  ^1^ Adjusted for maternal occupation, place of birth, distance from the road, moved house, season.  ^2^ Never married/ divorced/widowed  ^3^ At due date of vaccination | | | | |
