# Supplementary material for: Predictors of Uptake and Timeliness of Newly Introduced Pneumococcal and Rotavirus Vaccines, and of Measles Vaccine in Rural Malawi: A Population Cohort Study
Source: PLoS One. 2016 May 6;11(5):e0154997. doi: 10.1371/journal.pone.0154997 (PMC4859501; doi:10.1371/journal.pone.0154997)
Supplement: S6 Table — (DOCX) [file pone.0154997.s006.docx]

| **S6 Table. Survival analysis of predictors of timeliness of rotavirus vaccination** | | | | |
| --- | --- | --- | --- | --- |
| Variable | N | Median delay in days (IQR) | Crude hazard ratio (95%CI) | Adjusted hazard ratio^1^ (95%CI) |
| Gender |  |  |  |  |
| Female | 197 | 45 (20-101) | 1 | 1 |
| Male | 190 | 41 (18-116) | 1.05 (0.84-1.30) | 1.06 (0.85-1.32) |
| Mother’s age |  |  |  |  |
| <20 | 58 | 33 (16-87) | 1 | 1 |
| 20-29 | 222 | 46 (21-100) | 0.87 (0.64-1.18) | 1.04 (0.75-1.44) |
| 30-39 | 98 | 44 (19-145) | 0.81 (0.57-1.14) | 0.97 (0.68-1.39) |
| ≥ 40 | 8 | 6 (5-16) | 2.91 (1.38-6.14) | 3.47 (1.63-7.41) |
| Mother’s education |  |  |  |  |
| <5 years primary | 27 | 46 (19-*) | 1 | 1 |
| >= 5 years primary | 255 | 48 (22-113) | 1.34 (0.84-2.14) | 1.07 (0.66-1.72) |
| Secondary / tertiary | 104 | 30 (11-68) | 1.77 (1.08-2.90) | 1.36 (0.82-2.24) |
| Mother’s marital status |  |  |  |  |
| Married | 345 | 45 (19-110) | 1 | 1 |
| Unmarried^2^ | 41 | 31 (13-67) | 1.24 (0.88-1.75) | 1.22 (0.85-1.74) |
| Mother mobile phone personal use |  |  |  |  |
| No | 317 | 46 (20-113) | 1 | 1 |
| Yes | 65 | 29 (13-73) | 1.32 (0.99-1.75) | 1.25 (0.94-1.66) |
| Mother’s occupation |  |  |  |  |
| Farming | 356 | 45 (19-104) | - | - |
| Other | 27 | 28 (12-67) | 1.23 (0.81-1.86) | 1.15 (0.76-1.77) |
| Orphanhood |  |  |  |  |
| Both parents alive | 379 | 42 (18-104) | 1 | 1 |
| Father died | 4 | 61 (13-176) | 0.62 (0.20-1.93) | 0.71 (0.23-2.23) |
| Mother died | 2 | 35 (35-86) | 1.09 (0.27-4.37) | 1.13 (0.27-4.65) |
| Both died | - |  | - | - |
| Place of birth |  |  |  |  |
| Health centre | 358 | 42 (18-101) | 1 | 1 |
| Home / TBA / other | 24 | 37 (30-215) | 0.76 (0.47-1.22) | 0.92 (0.57-1.50) |
| Housing standard |  |  |  |  |
| 1 (lowest) | 41 | 62 (29-169) | 1 | 1 |
| 2 | 124 | 49 (22-145) | 1.09 (0.74-1.60) | 0.86 (0.58-1.29) |
| 3 | 52 | 34 (16-101) | 1.28 (0.82-2.01) | 1.00 (0.62-1.59) |
| 4 (highest) | 38 | 26 (12-65) | 1.73 (1.07-2.78) | 1.22 (0.74-2.01) |
| Household size (persons) |  |  |  |  |
| <4 | 66 | 35 (21-81) | 1 | 1 |
| 4-6 | 215 | 49 (19-119) | 0.85 (0.63-1.13) | 1.09 (0.76-1.55) |
| ≥ 7 | 106 | 31 (18-81) | 1.01 (0.73-1.41) | 1.49 (1.02-2.18) |
| Number of children <5 years in household |  |  |  |  |
| 1 | 144 | 31 (13-73) | 1 | 1 |
| 2 | 220 | 47 (23-133) | 0.72 (0.57-0.90) | 0.80 (0.64-1.00) |
| ≥ 3 | 23 | 64 (19-*) | 0.52 (0.31-0.87) | 0.55 (0.33-0.92) |
| Distance to road (km) |  |  |  |  |
| <1 | 290 | 33 (16-77) | 1 | 1 |
| 1-1.49 | 44 | 73 (44-215) | 0.51 (0.36-0.74) | 0.53 (0.37-0.75) |
| ≥ 1.5 | 52 | 72 (36-*) | 0.47 (0.33-0.67) | 0.50 (0.35-0.71) |
| Distance to clinic (km) |  |  |  |  |
| <1 | 277 | 44 (18-89) | 1 | 1 |
| 1-1.49 | 82 | 38 (22-134) | 0.88 (0.67-1.14) | 0.95 (0.73-1.25) |
| ≥ 1.5 | 28 | 46 (19-*) | 0.61 (0.39-0.98) | 0.71 (0.44-1.14) |
| Moved house |  |  |  |  |
| No | 374 | 41 (19-104) | 1 | 1 |
| Yes | 13 | 57 (21-97) | 0.80 (0.43-1.52) | 0.96 (0.51-1.82) |
| Season^3^ |  |  |  |  |
| Dry | 138 | 42 (18-97) | 1 | 1 |
| Rainy | 249 | 41 (19-113) | 0.92 (0.74- 1.15) | 0.96 (0.77- 1.20) |
| TBA = Traditional Birth Attendant  ^1^Adjusted for distance to road and number of children <5 in the household  ^2^ Never married/divorced/widowed  ^3^ At time of due date of vaccination  * No 75^th^ percentile delay reported: >25% not vaccinated | | | | |
