# Supplementary material for: Predictors of Uptake and Timeliness of Newly Introduced Pneumococcal and Rotavirus Vaccines, and of Measles Vaccine in Rural Malawi: A Population Cohort Study
Source: PLoS One. 2016 May 6;11(5):e0154997. doi: 10.1371/journal.pone.0154997 (PMC4859501; doi:10.1371/journal.pone.0154997)
Supplement: S7 Table — (DOCX) [file pone.0154997.s007.docx]

| **S7 Table. Survival analysis of predictors of timeliness of measles vaccination** | | | | |
| --- | --- | --- | --- | --- |
| Variable | N | Median delay in days (IQR) | HR (95%CI) | aHR^1^ (95%CI) |
| Gender |  |  |  |  |
| Female | 898 | 23 (5-52) | 1 | 1 |
| Male | 900 | 22 (4-53) | 1.03 (0.94, 1.14) | 1.00 (0.91, 1.11) |
| Mother’s age |  |  |  |  |
| <20 | 279 | 22 (3-49) | 1 | 1 |
| 20-29 | 982 | 22 (4-50) | 0.97 (0.85, 1.11) | 1.03 (0.89, 1.19) |
| 30-39 | 477 | 27 (6-60) | 0.87 (0.75, 1.02) | 0.95 (0.81, 1.12) |
| ≥ 40 | 58 | 29 (10-56) | 0.76 (0.56, 1.03) | 0.83 (0.61, 1.13) |
| Mother’s education |  |  |  |  |
| <5 years primary | 126 | 28 (6-83) | 1 | 1 |
| >= 5 years primary | 1163 | 23 (5-54) | 1.33 (1.09, 1.62) | 1.30 (1.06, 1.59) |
| Secondary / tertiary | 508 | 20 (4-48) | 1.47 (1.19, 1.81) | 1.34 (1.08, 1.67) |
| Mother’s marital status |  |  |  |  |
| Married | 1605 | 23 (4-52) | 1 | 1 |
| Unmarried^2^ | 190 | 20 (6-55) | 0.98 (0.84, 1.15) | 0.91 (0.77, 1.07) |
| Mother mobile phone personal use |  |  |  |  |
| No | 1328 | 24 (5-54) | 1 | 1 |
| Yes | 223 | 20 (4-54) | 1.03 (0.89, 1.19) | 0.96 (0.82, 1.12) |
| Mother’s occupation |  |  |  |  |
| Farming | 1646 | 23 (5-55) | 1 | 1 |
| Other | 123 | 16 (2-32) | 1.45 (1.21, 1.75) | 1.37 (1.12, 1.66) |
| Orphanhood |  |  |  |  |
| Both parents alive | 1760 | 23 (4-52) | 1 | 1 |
| Father died | 20 | 16 (6-57) | 1.04 (0.67, 1.62) | 1.07 (0.68, 1.69) |
| Mother died | 5 | 34 (22-44) | 1.25 (0.52, 3.00) | 1.18 (0.44, 3.17) |
| Both died | - |  | - | - |
| Place of birth |  |  |  |  |
| Health centre | 1604 | 22 (4-50) | 1 | 1 |
| Home / TBA / other | 160 | 27 (4-74) | 0.77 (0.65, 0.92) | 0.82 (0.69, 0.98) |
| Housing standard |  |  |  |  |
| 1 (lowest) | 258 | 27 (4-62) | 1 | 1 |
| 2 | 617 | 26 (5-55) | 1.04 (0.90, 1.21) | 1.00 (0.86, 1.17) |
| 3 | 283 | 22 (5-59) | 1.09 (0.91, 1.30) | 1.00 (0.84, 1.20) |
| 4 (highest) | 249 | 19 (4-45) | 1.23 (1.03, 1.47) | 1.06 (0.88, 1.29) |
| Household size (persons) |  |  |  |  |
| <4 | 354 | 21 (4-48) | 1 | 1 |
| 4-6 | 953 | 22 (4-50) | 0.94 (0.83, 1.07) | 1.05 (0.90-1.23) |
| ≥ 7 | 491 | 28 (6-61) | 0.82 (0.71, 0.94) | 0.94 (0.79-1.11) |
| Number of children <5y in household |  |  |  |  |
| 1 | 742 | 20 (4-48) | 1 | 1 |
| 2 | 925 | 25 (5-56) | 0.86 (0.78, 0.95) | 0.89 (0.80, 0.98) |
| ≥ 3 | 131 | 26 (4-62) | 0.82 (0.67, 0.99) | 0.83 (0.68, 1.00) |
| Distance to road (km) |  |  |  |  |
| <1 | 1362 | 21 (4-49) | 1 | 1 |
| 1-1.49 | 259 | 23 (6-58) | 0.89 (0.78, 1.03) | 0.96 (0.83-1.10) |
| ≥ 1.5 | 177 | 32 (11-73) | 0.78 (0.66, 0.92) | 0.85 (0.71-1.01) |
| Distance to clinic (km) |  |  |  |  |
| <1 | 1251 | 21 (4-50) | 1 | 1 |
| 1-1.49 | 375 | 25 (7-54) | 0.92 (0.81, 1.03) | 0.95 (0.84, 1.07) |
| ≥ 1.5 | 172 | 27 (4-77) | 0.80 (0.67, 0.94) | 0.86 (0.72, 1.02) |
| Moved house |  |  |  |  |
| No | 1723 | 23 (4-52) | 1 | 1 |
| Yes | 75 | 27 (6-74) | 0.87 (0.68, 1.11) | 0.88 (0.69, 1.13) |
| Season^3^ |  |  |  |  |
| Dry | 1042 | 22 (4-55) | 1 | 1 |
| Rainy | 756 | 24 (5-49) | 0.97 (0.87- 1.07) | 0.96 (0.87- 1.06) |
| ^1^ Adjusted for maternal education, maternal occupation, number of children <5 years in the household and place of birth.  ^2^ Never married/divorced/widowed  ^3^ At due date of measles vaccination | | | | |
