# Supplementary material for: Predictors of Uptake and Timeliness of Newly Introduced Pneumococcal and Rotavirus Vaccines, and of Measles Vaccine in Rural Malawi: A Population Cohort Study
Source: PLoS One. 2016 May 6;11(5):e0154997. doi: 10.1371/journal.pone.0154997 (PMC4859501; doi:10.1371/journal.pone.0154997)
Supplement: S8 Table — (DOCX) [file pone.0154997.s008.docx]

| **S8 Table. Literature reporting risk factors for low vaccine uptake and/or late vaccination in sub-Saharan countries** | | | | |
| --- | --- | --- | --- | --- |
| **Reference** | **Data source** | **Country** | **Rural/urban** | **Risk factors for low vaccine uptake and/or late vaccination** |
| Abadura SA et al, 2015 [[1](#_ENREF_1)] | DHS | Ethiopia | Both | -non-facility birth  -low maternal education  -low socio-economic status  -resident in rural area  -increasing number of children <5 years in the household |
| Babalola S, 2008 [[2](#_ENREF_2)] | Household survey | Nigeria | Both | -non-facility birth  -maternal age <20 years  -low maternal education  -low socio-economic status |
| Babirye JN et al, 2012 [[3](#_ENREF_3)] | Cross-sectional survey | Kampala, Uganda | Urban | -non-facility birth  -increasing number of children per woman  -household poverty |
| Bosch-Capblanch X et al, 2012 [[4](#_ENREF_4)] | Review of DHS | 96 low & middle income countries including Malawi | Both | -household poverty  -low education status of caregiver and caregiver’s partner  -caregivers not received tetanus toxoid vaccine |
| Canavan ME et al, 2014 [[5](#_ENREF_5)] | Review of DHS in Africa | Burundi, Ethiopia, Kenya, Rwanda, Tanzania, and Uganda | Both | -non-facility birth |
| Fadness LT et al, 2011 [[6](#_ENREF_6)] | Secondary analysis data from cluster randomised controlled trial | Uganda | Both | -low maternal education |
| Fadness LT et al, 2011 [[7](#_ENREF_7)] | Secondary analysis data from cluster randomised controlled trial | South Africa | Both | -low maternal education  -non-facility birth  -resident in rural area |
| Favin M et al, 2012 [[8](#_ENREF_8)] | Review of grey literature | Global (53.9% African) | Both | -long distances to health facilities  -low socio-economic status  -low parental educational status |
| Glatman-Freedman A & Nichols K, 2012 [[9](#_ENREF_9)] | Review | Low, middle & high income countries | Both | -low socio-economic status  -low parental education particularly maternal education.  -long distances to health facilities |
| Gram L et al, 2014 [[10](#_ENREF_10)] | Secondary analysis of HDSS | Ghana | Rural | -resident in rural area  -low maternal education  -low socio-economic status |
| Jahn A et al, 2008 [[11](#_ENREF_11)] | HDSS | Malawi | Rural | -non-facility birth  -low parental education  -long distances to health facilities  -low socio-economic status |
| Jani JV et al, 2007 [[12](#_ENREF_12)] | Cross-sectional survey | Mozambique | Rural | -long distances to health facilities  -low maternal education  -non-facility birth |
| Le Polain de Waroux O et al, 2012 [[13](#_ENREF_13)] | Cluster survey | Southern Tanzania | Rural | -long distances to health facilities  -household poverty  -low maternal education  -rainy season |
| Malawi Demographic and Health Survey 2010 [[14](#_ENREF_14)] | DHS | Malawi | 90% of the population live in rural areas. | -resident in urban area  -low maternal education  -low socio-economic status |
| Munthali AA, 2007 [[15](#_ENREF_15)] | DHS 1992, 1996, 2000, 2004 | Malawi | Both | -low maternal education  -resident in rural areas  -increased child birth order |
| Odutola A et al, 2015 [[16](#_ENREF_16)] | Cross sectional survey | Western Gambia | Both | -increased child birth order  -non-facility birth |
| Payne S et al, 2014 [[17](#_ENREF_17)] | HDSS | Gambia | Both | -resident in urban areas  -ethnicity (Mandinka) |
| Rainey JJ et al, 2011 [[18](#_ENREF_18)] | Systematic literature review | Global literature published between 1999-2009 | Both | -low maternal education  -low socio-economic status  -long distances to health facilities  -high costs (direct and indirect)  -fear of adverse events |
| Schoeps A et al, 2013 [[19](#_ENREF_19)] | HDSS | Burkina Faso | Both | -low maternal education  -low socio-economic status  -dry season at birth  -long distances to health facilities |
| Wysonge CS et al, 2012 [[20](#_ENREF_20)] | DHS | 24 countries in sub-Saharan Africa | Both | -low maternal education  -low paternal education  -low socio-economic status  -resident in urban areas  -countries with high fertility rates |

Abbreviations: DHS, Demographic and Health Survey, HDSS, Health and Demographic Surveillance System

**References**

1. Abadura SA, Lerebo WT, Kulkarni U, Mekonnen ZA. Individual and community level determinants of childhood full immunization in Ethiopia: a multilevel analysis. BMC Public Health. 2015;15(1):972. doi: 10.1186/s12889-015-2315-z.

2. Babalola S. Determinants of the uptake of the full dose of diphtheria-pertussis-tetanus vaccines (DPT3) in Northern Nigeria: a multilevel analysis. Matern Child Health J. 2009;13(4):550-8. doi: 10.1007/s10995-008-0386-5.

3. Babirye JN, Engebretsen IM, Makumbi F, Fadnes LT, Wamani H, Tylleskar T, et al. Timeliness of childhood vaccinations in Kampala Uganda: a community-based cross-sectional study. PLoS One. 2012;7(4):e35432. doi: 10.1371/journal.pone.0035432.

4. Bosch-Capblanch X, Banerjee K, Burton A. Unvaccinated children in years of increasing coverage: how many and who are they? Evidence from 96 low- and middle-income countries. Trop Med Int Health. 2012;17(6):697-710. doi: 10.1111/j.1365-3156.2012.02989.x.

5. Canavan ME, Sipsma HL, Kassie GM, Bradley EH. Correlates of complete childhood vaccination in East African countries. PLoS One. 2014;9(4):e95709. doi: 10.1371/journal.pone.0095709.

6. Fadnes LT, Nankabirwa V, Sommerfelt H, Tylleskar T, Tumwine JK, Engebretsen IM. Is vaccination coverage a good indicator of age-appropriate vaccination? A prospective study from Uganda. Vaccine. 2011;29(19):3564-70. doi: 10.1016/j.vaccine.2011.02.093.

7. Fadnes LT, Jackson D, Engebretsen IM, Zembe W, Sanders D, Sommerfelt H, et al. Vaccination coverage and timeliness in three South African areas: a prospective study. BMC Public Health. 2011;11:404. doi: 10.1186/1471-2458-11-404.

8. Favin M, Steinglass R, Fields R, Banerjee K, Sawhney M. Why children are not vaccinated: a review of the grey literature. Int Health. 2012;4(4):229-38. doi: 10.1016/j.inhe.2012.07.004.

9. Glatman-Freedman A, Nichols K. The effect of social determinants on immunization programs. Hum Vaccin Immunother. 2012;8(3):293-301. doi: 10.4161/hv.19003.

10. Gram L, Soremekun S, ten Asbroek A, Manu A, O'Leary M, Hill Z, et al. Socio-economic determinants and inequities in coverage and timeliness of early childhood immunisation in rural Ghana. Trop Med Int Health. 2014;19(7):802-11. doi: 10.1111/tmi.12324.

11. Jahn A, Floyd S, Mwinuka V, Mwafilaso J, Mwagomba D, Mkisi RE, et al. Ascertainment of childhood vaccination histories in northern Malawi. Trop Med Int Health. 2008;13(1):129-38. doi: 10.1111/j.1365-3156.2007.01982.x.

12. Jani JV, De Schacht C, Jani IV, Bjune G. Risk factors for incomplete vaccination and missed opportunity for immunization in rural Mozambique. BMC Public Health. 2008;8:161. doi: 10.1186/1471-2458-8-161.

13. Le Polain de Waroux O, Schellenberg JR, Manzi F, Mrisho M, Shirima K, Mshinda H, et al. Timeliness and completeness of vaccination and risk factors for low and late vaccine uptake in young children living in rural southern Tanzania. Int Health. 2013;5(2):139-47. doi: 10.1093/inthealth/iht006.

14. National Statistical Office (NSO) and ICF Macro. Malawi Demographic and Health Survey 2010. Zomba, Malawi, and Calverton, Maryland, USA: NSO and ICF Macro. 2011.

15. Munthali AC. Determinants of vaccination coverage in Malawi: evidence from the demographic and health surveys. Malawi Med J. 2007;19(2):79-82.

16. Odutola A, Afolabi MO, Ogundare EO, Lowe-Jallow YN, Worwui A, Okebe J, et al. Risk factors for delay in age-appropriate vaccinations among Gambian children. BMC Health Serv Res. 2015;15:346. doi: 10.1186/s12913-015-1015-9.

17. Payne S, Townend J, Jasseh M, Lowe Jallow Y, Kampmann B. Achieving comprehensive childhood immunization: an analysis of obstacles and opportunities in The Gambia. Health Policy Plan. 2014;29(2):193-203. doi: 10.1093/heapol/czt004.

18. Rainey JJ, Watkins M, Ryman TK, Sandhu P, Bo A, Banerjee K. Reasons related to non-vaccination and under-vaccination of children in low and middle income countries: findings from a systematic review of the published literature, 1999-2009. Vaccine. 2011;29(46):8215-21. doi: 10.1016/j.vaccine.2011.08.096.

19. Schoeps A, Ouedraogo N, Kagone M, Sie A, Muller O, Becher H. Socio-demographic determinants of timely adherence to BCG, Penta3, measles, and complete vaccination schedule in Burkina Faso. Vaccine. 2013;32(1):96-102. doi: 10.1016/j.vaccine.2013.10.063.

20. Wiysonge CS, Uthman OA, Ndumbe PM, Hussey GD. Individual and contextual factors associated with low childhood immunisation coverage in sub-Saharan Africa: a multilevel analysis. PLoS One. 2012;7(5):e37905. doi: 10.1371/journal.pone.0037905.
